# Evaluation of anti-rollback systems in manual wheelchairs: muscular activity and upper limb kinematics during propulsion

**DOI:** 10.1038/s41598-022-21806-z

**Published:** 2022-11-09

**Authors:** Bartosz Wieczorek, Mateusz Kukla, Łukasz Warguła, Marcin Giedrowicz, Dominik Rybarczyk

**Affiliations:** 1 Faculty of Mechanical Engineering, Poznan University of Technology, Piotrowo 3 St., 424 BM, 61-139 Poznań, Poland; 2 Faculty of Architecture, Poznan University of Technology, Poznań, Poland

**Keywords:** Biomedical engineering, Musculoskeletal system

## Abstract

Self-propelling a wheelchair up a hill requires intense muscular effort and introduces the risk of the wheelchair rolling down. The purpose of this paper was to assess the user's muscular activity during ramp climbing. Tests were carried out on a group of 10 subjects who had to propel a wheelchair up a standardized wheelchair ramp. Basic parameters of upper limb kinematics were measured to determine the total push-rim rotation angle. This was 105.91° for a wheelchair with a stiff anti-rollback system, 99.39° for a wheelchair without an anti-rollback system and 98.18° for a wheelchair with a flexible anti-rollback system. The upper limb muscle effort was measured at 55 ± 19% for the wheelchair without an anti-rollback system, 59 ± 19% for the wheelchair with a stiff anti-rollback system and 70 ± 46% for the wheelchair with a flexible anti-rollback system. The conducted research showed an increase in muscle effort while using anti-rollback systems. In the case of push-rim rotation angle, no significant differences in the value of the rotation angle were found.

## Introduction

Each wheelchair should ideally be tailored to the user's needs and to meet the user’s degree of disability and lifestyle. Of relevance is that the selection of a wheelchair best suited to the individual requirements allows achieving a greater degree of social inclusion. In order to adjust the wheelchair to the individual needs of its users^1^, it is equipped with various functional modules. Modifications to the drive system can be distinguished among the modules increasing the mobility of the wheelchair. The most popular modifications to the drive system of a classic manual wheelchair include a lever drive^2^ and a crank drive^3^. There is also a group of modifications that do not change the drive system. These utilize push-rims. Such modifications affect only the reduction of the force of resistance to motion. The most common types of such modifications are the FreeWheel attachment^4^ replacing the front wheels of the wheelchair with a single large diameter pneumatic wheel, and the reversing lock modules^5^ of the wheelchair to make it safer for use when on an incline. The reversing lock module is a new product introduced to the rehabilitation equipment market and still requires research to verify its functionality and improve its design.

Survey results suggest that disabled people want to use wheelchairs not only in their everyday life, but also for leisure in non-urban settings^6^. In order for the user to be able to use a wheelchair in such settings, the drive system has to generate a driving force that compensates for the higher movement resistance forces. In everyday life, the obstacles most frequently encountered by wheelchair users are architectural, which is especially true in the case of developing countries, where building standards in terms of accessibility for disabled people are often disregarded^7^. An example of a widely encountered type of obstacle that requires increased physical effort are ramps, the purpose of which is to replace stairs or to compensate for the difference in terrain elevation for wheelchair users. The guidelines of the U.S. State Department of Transportation of 2014 set the maximum slope of the ramp at 8.3% and its width at 1.22 m^8^. In the case of European standards, ramps used for elevation differences up to 0.5 m should have a slope angle up to 4.57°, while for elevation differences greater than 0.5 m, the angle should not exceed 3.42°^9^.

**Figure 1:**
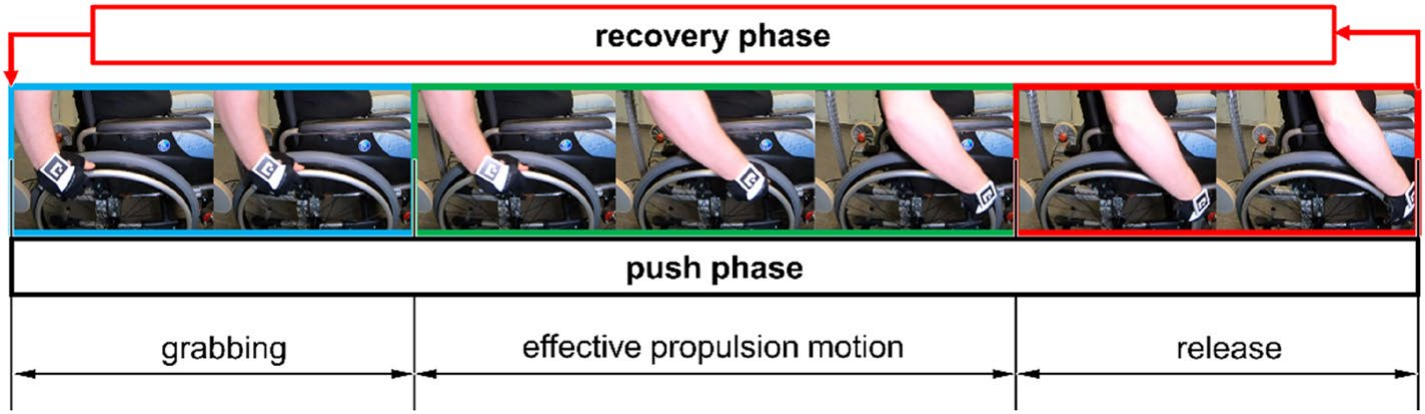
Schematic illustration showing the push phase broken down into individual stages.

Climbing up a ramp is one of the most difficult maneuvers performed by wheelchair users with motor disabilities. The difficulty stems from the need to continuously perform propulsion cycles without a rest. What is more, when moving up a ramp, the wheelchair is subject to additional motion resistance forces due to gravity^10–12^. These forces significantly increase the muscular effort required of the user compared to the effort when travelling on a flat surface. The propulsion phases are divided into push and recovery phases^13,14^. The push phase (Fig. 1) involves gripping the push-rims, the effective propulsion motion, and the release of the push-rims. In the push phase, the user prepares to grip the push-rims by positioning his/her hands along them and adjusting the linear speed of the hands to the tangential velocity of the push-rims. This constitutes a buffer stage that ends the recovery phase and starts the push phase. During the effective propulsion motion (EPM) stage, the hand holds the push-rim and its trajectory is the same as the shape of the push-rim. In the course of this move, muscle force is transferred to the propulsion system of the wheelchair and converted into driving force. The last stage of the push phase is the release of the push-rims. During this action, the hand grip is released and the speed of the hand changes in relation to the tangential velocity of the push-rims. The release constitutes a buffer stage that ends the push phase and starts the recovery phase. The return phase gives the user full freedom of movement of the upper limb. Therefore, the main return phase has the greatest impact on the kinematics of the upper limb and the trajectory of the hand^15^. In addition, during this phase, when climbing a ramp, there is a risk of the wheelchair rolling down. Research indicates that the pattern of hand trajectory is influenced by such factors as the frequency of propulsion phases and the total angle of hand to rim contact during the push phase^16,17^.

Movement up a ramp is characterized by an extended hand movement trajectory, an increased frequency of push phases and an increased muscle force converted into the wheelchair driving force. These parameters significantly differ from those typical of movement along a horizontal surface. The increased muscular force supplied to the propulsion system translates into an increase in mechanical energy^14,18^ required to overcome the motion resistance. Increased frequency of push phases eliminates the risk of the wheelchair rolling down the ramp during the return phase. It also determines possible hand movement trajectories^19^. As a result, the individual propulsion technique that compensates for the degree of motor disability of a given wheelchair user may be disturbed. The pattern of hand movement trajectory is also affected by the need to maximize the angle of hand to rim contact during the push phase. Available literature on the subject indicates limit values of hand to hand-rim contact angle during the push phase ranging from 100.3° to 110.3° depending on the velocity of wheelchair movement^17^. Published research shows that in the case of increased motion resistance typical of ramp climbing, a semicircular trajectory, whereby the upper limb moves close to the push-rims during the return phase, is most effective^20^. Ramp ascent not only affects the upper limb kinematics and hand trajectory, but also the maximum voluntary contraction (MVC)^21^ due to increased demand for muscle-generated force. Increased muscular activity resulting from the need to generate muscle force compensating for motion resistance during ramp ascent also translates into increased risk of upper limb skeletal muscle injuries^22^. Vulnerability to such injuries is the greatest in the case of wheelchairs using conventional push-rim propulsion systems, which require the stimulation of the largest number of muscle groups and may lead to various injuries of the skeletal and muscular system, e.g. shoulder joint degeneration^23^.

The mentioned correlations lead to a conclusion that conventional propulsion systems used when moving in challenging terrain, such as ramps, may cause pain and injury to the upper limb due to increased demand for muscle force^24^. Consequently, there is a need for innovative propulsion systems and wheelchairs that would compensate for physical limitations when overcoming terrain obstacles^25–28^. Such solutions should require from the user, physical activity, while compensating for his or her physical limitations. The main purpose of this paper was to assess the user's muscular activity and kinematics of upper limb movement during ramp ascent in a push-rim wheelchair equipped with various ramp assist modifications designed to improve the conventional manual drive system. A secondary purpose of the paper was to verify which variant of the anti-rollback system is better and should be adopted for design and construction.

## Material and method

### Anti-rollback system and measuring apparatus

**Figure 2:**
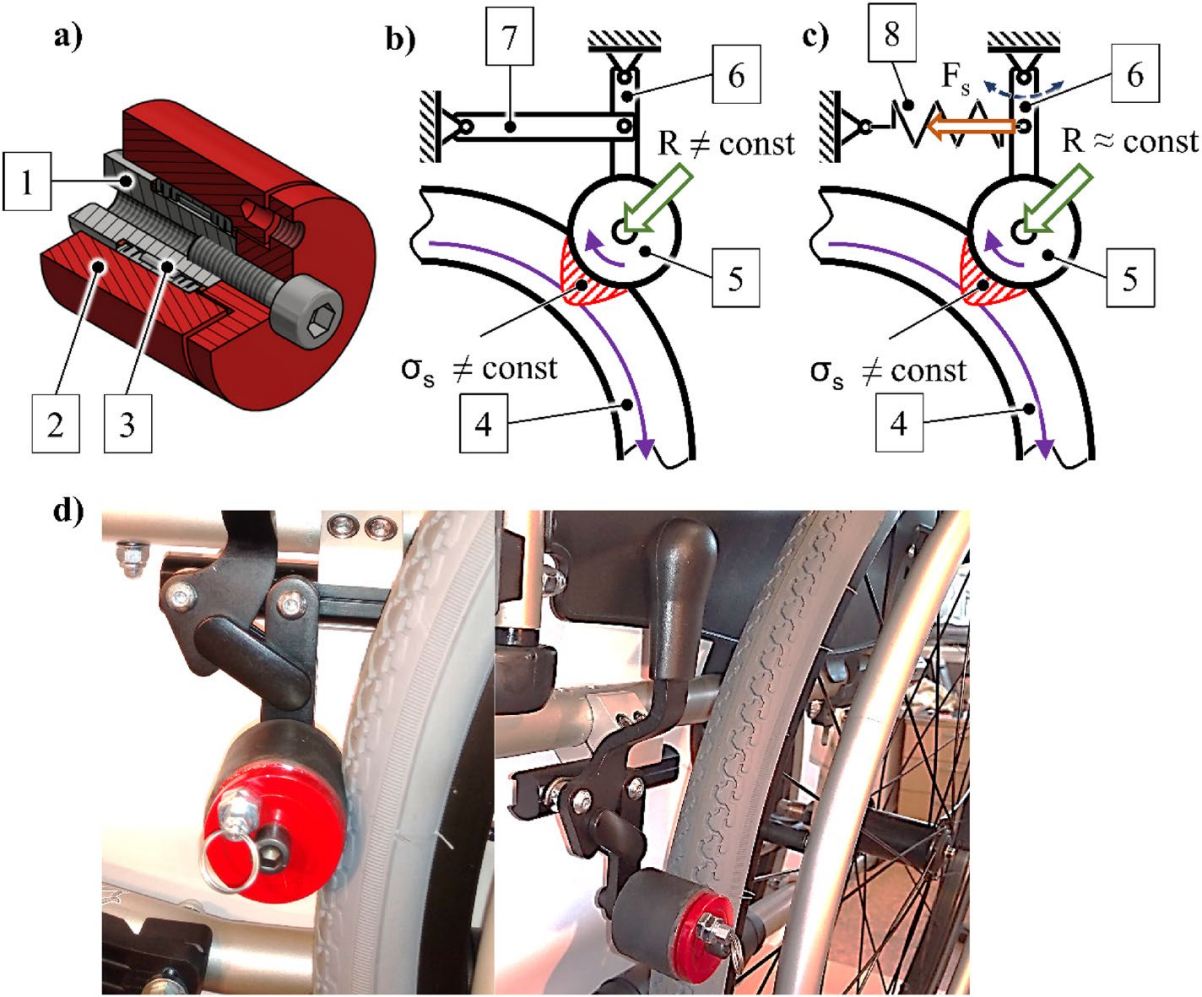
Anti-rollback system (**a**) coupling with the drive wheel by means of a rigid body (**b**) and elastic body (**c**), implementation of a rigid body variant (**d**); where: 1—central axle, 2—anti-rollback roller, 3—one-way clutch, 4—pneumatic drive wheel, 5—anti-rollback system, 6—articulated link, 7—rigid beam, 8—tension spring.

The research was conducted using a semi-active push-rim wheelchair Vermeiren v300. The wheelchair was equipped with two variants of the anti-rollback system^29,30^. Regardless of the variant, each anti-rollback system (Fig. 2a) consisted of a central axis with an anti-rollback roller fixed by means of a one-way clutch. The use of a one-way clutch means that the anti-rollback roller can only turn in the opposite direction to that of the wheelchair's drive wheel. Reverse rotation is blocked. By using this design feature and the frictional coupling of the wheel of the wheelchair with the anti-rollback roller, the reverse movement of the wheelchair is prevented.

Friction coupling is the result of the clamping force R applied to the anti-rollback roller. As a result of the force R, deformation occurs in the pneumatic tire of the drive wheel^31,32^, causing surface tension σ_s_^33,34^. Adopting a simplified model, it can be assumed that under static conditions, the clamping force R is constant and proportional to the surface tension (1).

1 $$R = \sigma_{s} A,$$

where: R—clamping force of the anti-rollback roller, σ_s_—surface tension between the tire and the anti-rollback roller, A—contact area between the anti-rollback roller and the drive wheel tire.

The pneumatic drive wheels of the wheelchair travel on surfaces with different deformability levels, including transverse bumps such as thresholds and curbs. This causes dynamic deformation of the tires^35^ and, hence, changes in the tire pressure^36^. Changes in the pressure inside the drive wheel tires make the surface tension σ_s_ values to be variable. Due to dynamic changes in the position of the wheelchair user's body and when overcoming obstacles, the increase in pressure perceptibly increases rolling resistance caused by the coupling of the anti-rollback roller with the pneumatic drive wheel of the wheelchair.

Due to the coupling characteristics of the pneumatic drive wheel to the road surface and the anti-rollback roller, two ways of the anti-rollback system installation were taken into account in the test. In the stiff variant (ARS) (Fig. 2b, d), the articulated link ends with the anti-rollback system being fixed by a rigid beam. The stiff variant is characterized by variability of the clamping force R of the anti-rollback roller depending on the variations in the surface tension σ_s_. In the flexible variant (ARE) (Fig. 2c), a tension spring is used instead of a rigid beam. In this variant, the spring compensates for dynamic changes in surface tension σ_s_, keeping the clamping force R constant under certain operating conditions. Such variants were adopted in order to determine further directions of design development, not to determine the geometrical features of the device. In each test, the clamping force R was adopted individually for each subject. The criterion for the selection of the R force was maintaining the static equilibrium of the wheelchair with the user on the tested elevation.

**Figure 3:**
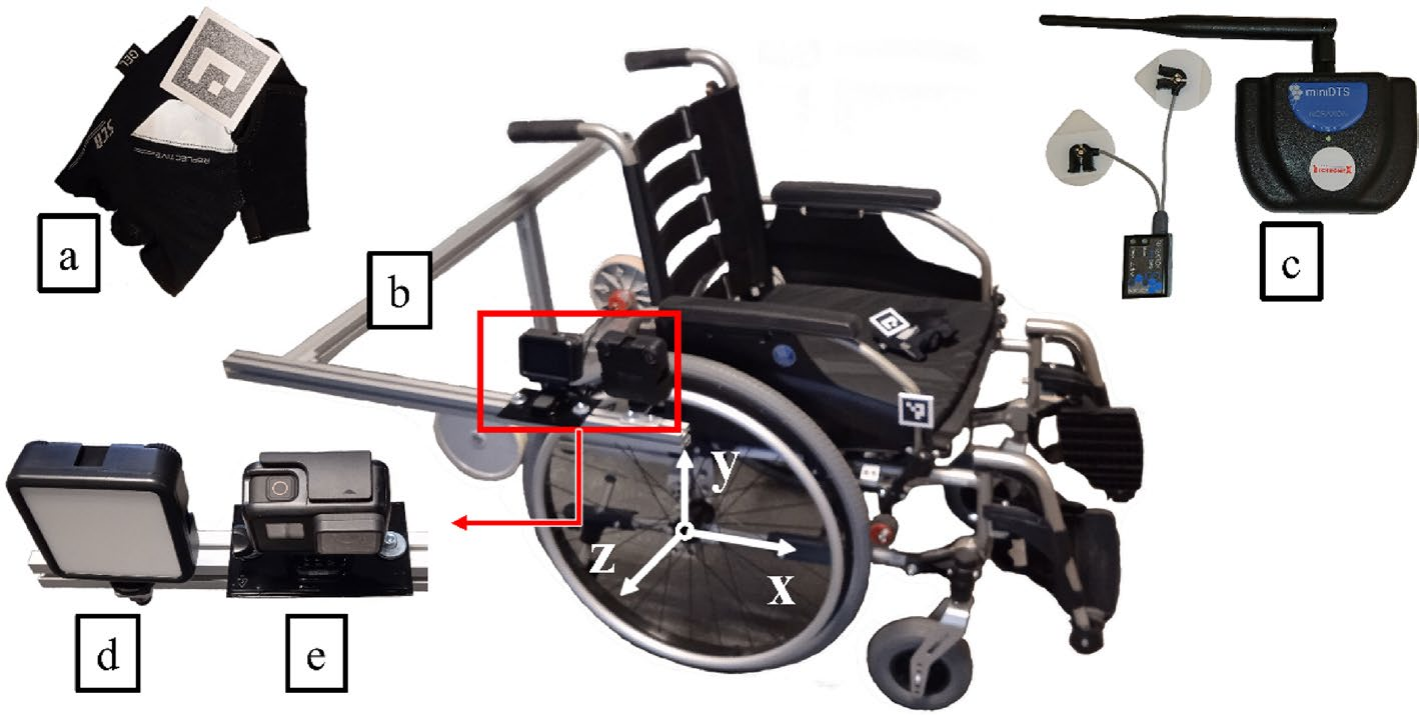
Measurement apparatus used for testing: (**a**)—AruCo marker, (**b**)—arm, (**c**)—EMG apparatus, (**d**)—illuminating lamp, (**e**)—camera.

A measurement system was connected to the tested wheelchair to assess limb mobility (Fig. 3). The system consisted of a GoPro HERO 7 camera (e) and an illuminating lamp (d) mounted on an arm (b) permanently connected to the wheelchair frame. The camera captured 960p quality video at 240 fps. The illuminating lamp generated between 200 and 1000 lumens. The camera recorded 50 mm by 50 mm AruCo markers (a). Surface electromyography EMG was measured using a Noraxon mini DTS device (c).

### Test method

**Figure 4:**
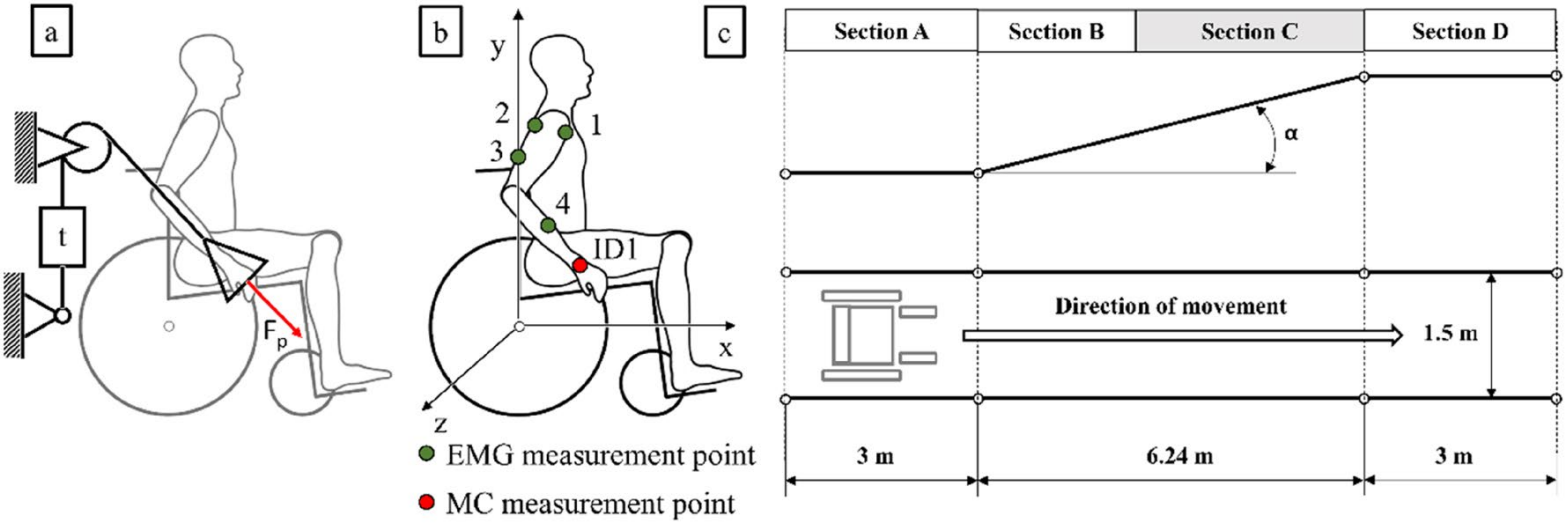
Schematic illustration of the push force measurement system (**a**), location of the motion capture and EMG measurement points (**b**), and the ramp used to perform the test (**c**). In this diagram: t—strain gauge force sensor, F_p_—push force, section A—the section used for wheelchair acceleration, section B—the section for which the measurement results were disregarded, section C—the section for which the measurement results were taken into account, section D—the final section where the wheelchair was stopped, α – the ramp inclination.

The test was performed in real conditions during the operation of the wheelchair in a closed room of a public building. The research was carried out on men aged 30 to 36 years. The examined persons suffered damage to the spine of the lumbar (L1–L5) or sacral (S1–S5) spine. The spine injury in this section does not affect the mobility of the upper limbs. Ten subjects (Table 1) classified according to their height, weight, age, maximum upper limb push force, and wheelchair experience, were examined. The measurement method for the push force was formalized in terms of methodology. A special stand was used on which the user, in a seated position, pushed a handle connected to a strain gauge towards his or her knee (Fig. 4a). The evaluation of the participants' experience was based on a five-point scale. This assessment was performed by the examined patients, taking into account the time of using the wheelchair, the variety of places of its use and the general confidence in moving about in a wheelchair. Each subject was familiarized with the test procedure and completed informed consent forms to participate in the research. The research and experimental protocols were positively evaluated by the Bioethical Commission at the Karol Marcinkowski Medical University in Poznan Poland, Resolution No. 1100/16 of 10 November 2016, under the guidance of Prof. MD Chęciński P. for the research team led by Ph.D. Wieczorek B. The authors obtained written consent of the examined person for the publication of research results with his participation. The data was presented in such a way as to ensure complete anonymity. The measurement method and data acquisition were carried out in accordance with the directives of the Bioethics Commission at the Karol Marcinkowski Medical University in Poznan Poland, which are in line with the guidelines of the Helsinki Declarations.

**Table 1:** Comparison of test subject anthropometric features and the level of experience in wheelchair operation. Mean values determined at 95% confidence interval (p = 0.05).

	Height	Weight	Age	Push force	Experience
	cm	kg	years	N	[−]
Subject MK	183	90	32	364	●●●●●
Subject MKA	179	88	33	322	●●●●●
Subject BW	175	110	31	298	●●●●○
Subject BWA	178	96	30	309	●●●○○
Subject LWA	171	93	33	306	●●●○○
Subject LW	173	87	32	296	●●●●○
Subject DRA	169	72	30	263	●●●○○
Subject DR	174	81	35	247	●●●●○
Subject MKB	188	74	36	291	●●●○○
Subject MKC	185	72	36	321	●○○○○
AVG	178 ± 4	86 ± 9	33 ± 2	302 ± 23	–

During the test, each subject's muscle activity (MA) of the four muscle groups and the kinematics of hand movement were monitored (Fig. 4a). Measurement of muscle activity was performed on four muscle groups: Deltoid muscle anterior (1) and posterior (2), Triceps brachii (3), Extensor carpi radialis longus (4). EMG measurement was monitored unilaterally, taking into account the asymmetries between the left and right limbs^37^, and the dominant limb was always used for measurement. The analysis of kinematics was limited to the analysis of hand movement (ID1) (Fig. 4b). During the test, the subject had to complete a track consisting of four sections (Fig. 4c): a horizontal section for wheelchair acceleration (section A), a ramp section with an inclination of α = 4.58° (section B, section C) and a horizontal section for wheelchair deceleration (section D). Full propulsion movements made at the final section of the ramp (section C) were selected for analysis in order to minimize the impact of the mechanical energy^38^ accumulated during the acceleration phase of the wheelchair on the first horizontal section (inertia) (section A).

**Figure 5:**
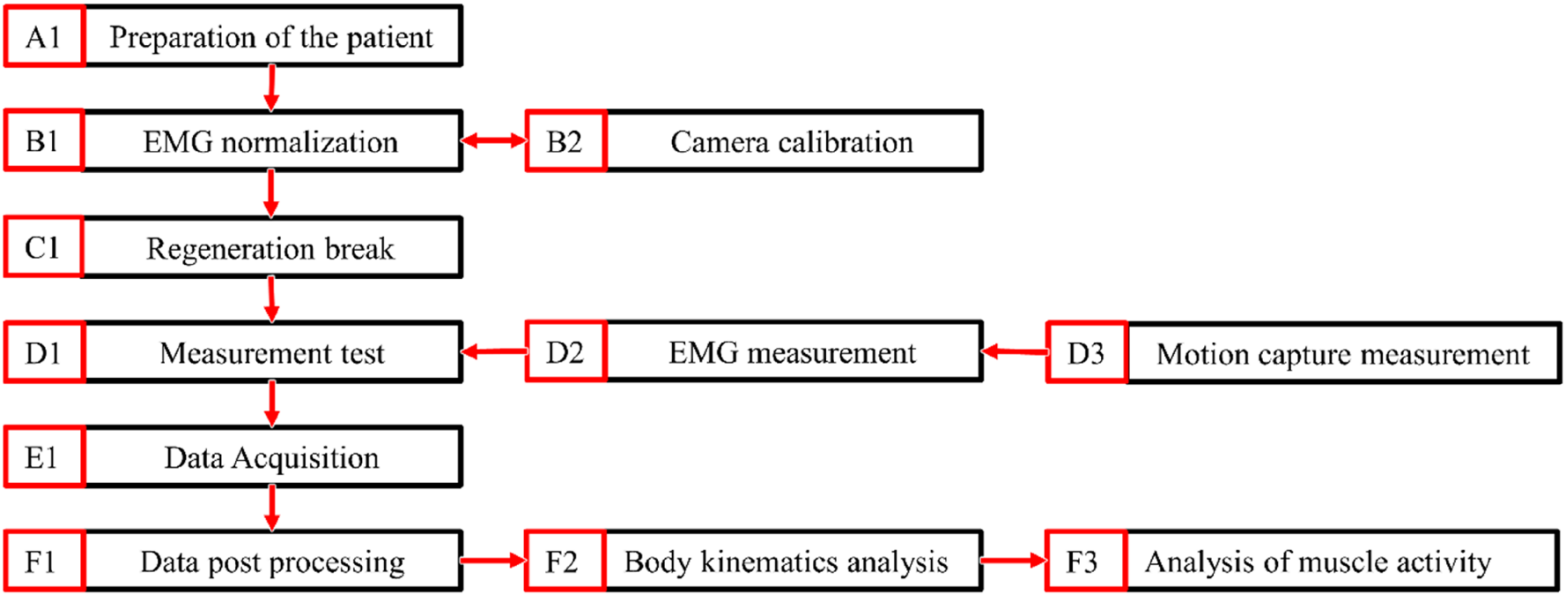
Block diagram of the performed measurement method.

Detailed methodology of the test is shown in the diagram (Fig. 5). The first subject was (A1), who was equipped with a set of electrodes for EMG measurements and a marker for motion capture recording. Subsequently, the process of EMG signal normalization (B1) and calibration of the camera with image processing software (B2) was performed. The procedure was carried out in accordance with the guidelines of the EMG camera manufacturer^39^. Its purpose was to determine the reference value for further calculations. A set of five dedicated exercises was carried out to test the maximum contraction of any muscle. This was selected on the basis of previous studies^21^. As part of the standardization exercises, users performed the following activities: lateral arm raise, lateral arm decline, forearm extension (elbows supported on thighs), wrist bend (underhand grip) and forearm flexion (elbows supported on thighs). The recorded data were successively normalized, taking the arithmetic mean of the amplitude of the highest signal segment with a constant duration of 1000 ms as the reference value. The normalization procedure was always followed by a regeneration break (C1) lasting 30 min. Subsequently, a measurement test (D1) was performed, which included an EMG measurement (D2) and a motion capture measurement of the hand (D3). Both measurements were performed simultaneously. At the end of each measurement, test data was collected (E1) and processed (F1). Hand kinematics (F2) and muscle activity of selected muscle groups (F3) was analyzed. After each measurement test (D1), the subject returned to the beginning of the track where he was allowed to rest for 10 min before the next round.

### Data processing procedures

**Figure 6:**
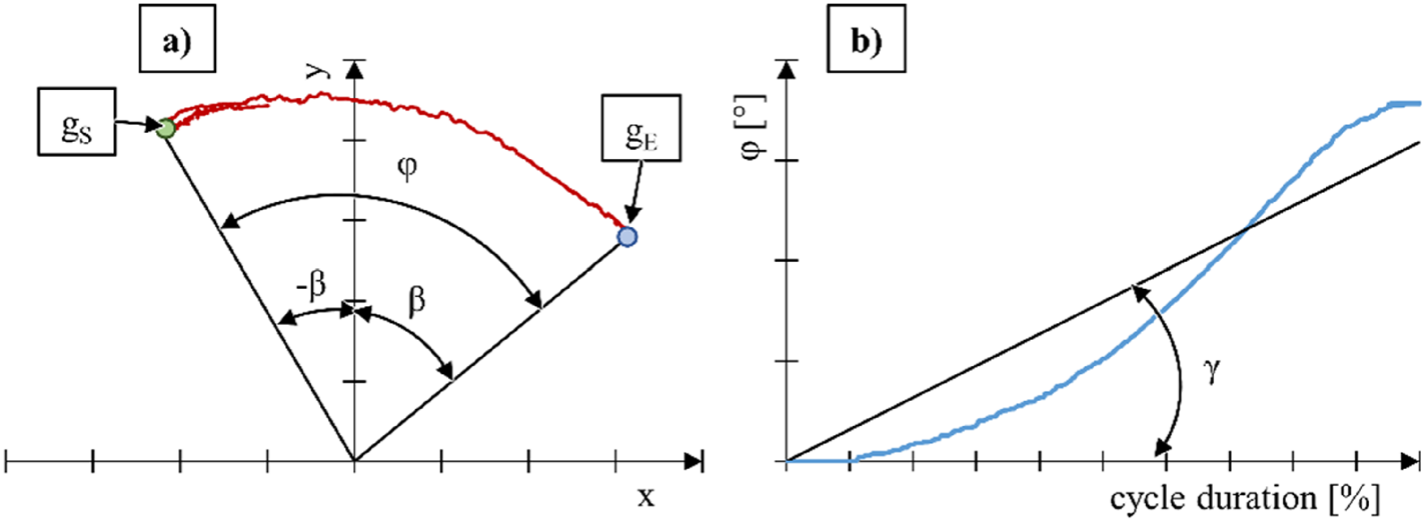
Graph representing hand motion trajectory (**a**) and the total angle of rotation of the drive wheel as a function of the percentage share of the total propulsion cycle (**b**). Here: g_S_—point of push-rim gripping, g_E_—point of push-rim release, γ—inclination of the trend line, β—angular position of the hand relative to the vertical axis, φ—total angle of rotation of the drive wheel.

Processing of the measurement data allowed obtaining information on hand kinematics and muscle activity (MA) of the upper limb. Hand kinematics was represented by the trajectory of hand motion during the propulsion cycle (Fig. 6a), which was used to determine the angular position of the hand relative to the vertical axis β and the total angle of rotation of the drive wheel φ as a function of the percentage share of the propulsion cycle total duration (Fig. 6b).

The initial angular position relative to the vertical axis of symmetry of the drive wheel was marked as − β (2), while the final position was designated as β (3).

2 $$- \beta = \cos^{ - 1} \left( {\frac{{g_{S}^{y} n}}{{\sqrt {g_{S}^{x2} + g_{S}^{y2} } + \sqrt n }}} \right)$$

3 $$\beta = \cos^{ - 1} \left( {\frac{{g_{E}^{y} n}}{{\sqrt {g_{E}^{x2} + g_{E}^{y2} } + \sqrt n }}} \right)$$

where: g_S_^x^—position of the g_S_ point on the horizontal axis, g_S_^y^—position of the g_S_ point on the vertical axis, g_E_^x^—position of the g_E_ point on the horizontal axis, g_E_^y^—position of the g_E_ point on the vertical axis, n—any positive real number.

**Figure 7:**
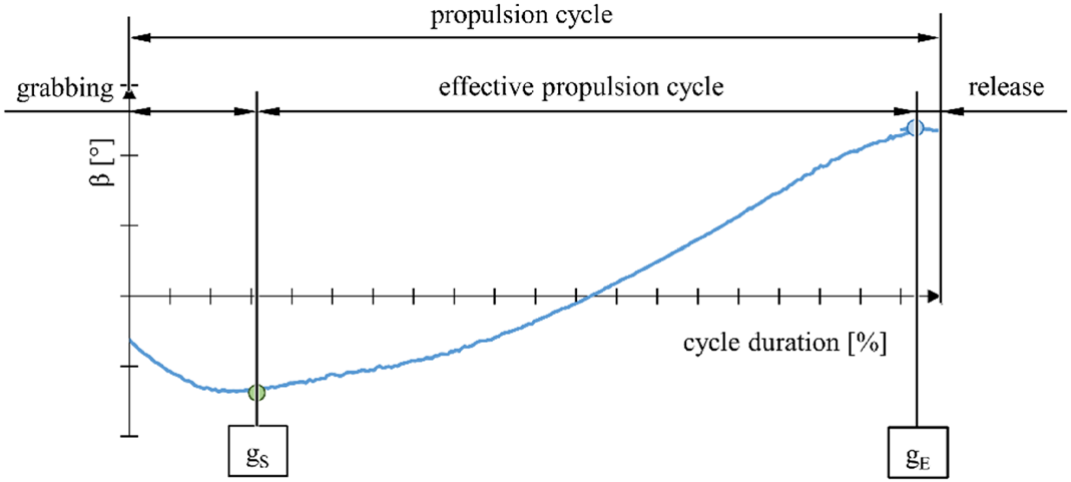
Graph representing angular position of the hand relative to the vertical axis as a function of the percentage share of the total propulsion cycle, with individual stages of the propulsion cycle identified, where: g_S_—push-rim gripping point, g_E_—push-rim release point.

The analysis of the variability of the hand position angle relative to the push-rims allowed for identification of individual stages of the propulsion cycle (Fig. 7) and determination of push-rim gripping (g_S_) and release points (g_E_). In order to determine these points, the hand angular position function β was used. The minimum of the function denoted push-rim gripping point (g_S_), while the maximum expressed push-rim release (g_E_).

As part of the analysis of the total angle of rotation of the drive wheel φ (4), the inclination of the trend line γ was calculated. The analysis of this parameter allowed assessing the impact of the examined technical solution on the increment of the push-rim rotation angle.

4 $$\varphi = \cos^{ - 1} \left( {\frac{{g_{S}^{E} g_{E}^{E} + g_{S}^{y} g_{E}^{y} }}{{\sqrt {g_{S}^{x2} + g_{S}^{y2} } + \sqrt {g_{E}^{x2} + g_{E}^{y2} } }}} \right)$$

**Figure 8:**
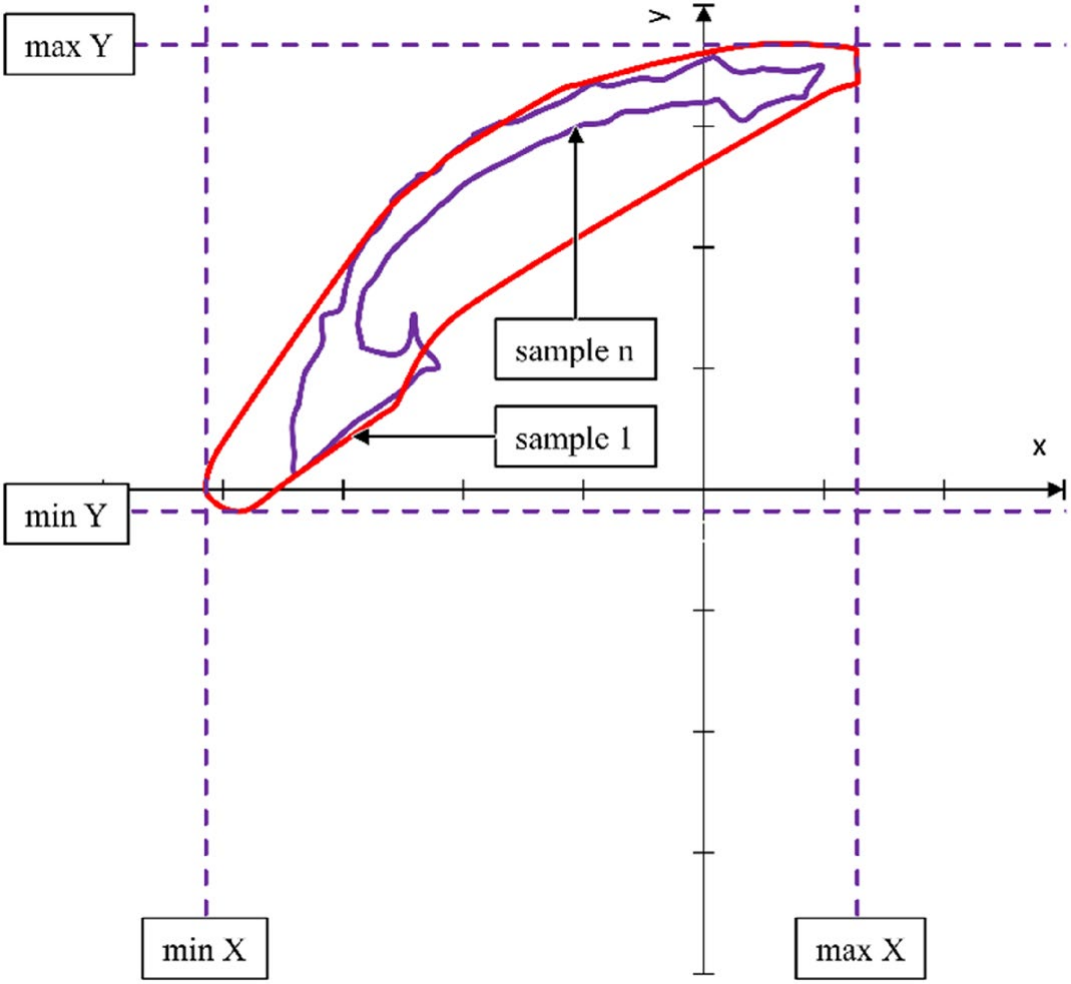
Graph representing hand position during wheelchair propulsion, where: sample 1 and sample n are the outline of the analyzed measurement sample consisting of five full propulsion cycles.

In addition, the motion capture measurement allowed analyzing the hand position when propelling different variants of the anti-rollback system (Fig. 8). For this purpose, three measurement tests consisting of five full propulsion cycles were conducted separately. On this basis, the outline of the point cloud was determined. To generate the outline, the alpha shape algorithm^39^ was used with the alpha coefficient in the range of 0.7—0.8. The analysis of the area occupied by the hand during propulsion focuses on ascertaining the length L (5) and the height H (6) of the field drawn by the hand.

5 $$L = \max X - \min X$$

6 $$H = \max Y - \min Y$$

In order to determine muscular activity (MA), a surface electromyography of the upper limb muscle was conducted using a Noraxon mini DTS surface electromyography device equipped with four measurement channels. The muscle activity signal was analyzed and recorded using Noraxon MR3 software. Muscle effort analysis was conducted for four muscles that are involved in the propulsion of the wheelchair: Deltoid muscle anterior (canal 1) and posterior (canal 2), Triceps brachii (canal 3), Extensor carpi radialis longus (canal 4). Before the actual measurement of muscle activity (during the ascent), each subject underwent a normalization procedure in accordance with the guidelines of the EMG device manufacturer^40^. Its purpose was to determine the reference value for further calculations. A set of five dedicated exercises was carried out to test the maximum contraction of any muscle. These exercises were selected on the basis of previous studies^21^. The recorded data were successively normalized, taking the arithmetic mean of the amplitude of the highest signal segment with a constant duration of 1000 ms as the reference value. Circular gel electrodes (⌀ 20 mm) were placed in the central part of the belly of the examined muscles. Measurements were performed at a frequency of 1500 Hz.

Normalization was performed on the same day as the actual test, with a break to allow the muscles to recover from the effort associated with the normalization procedure. After the regeneration break, the subjects performed the actual test on the ramp. The measured EMG signals were rectified and then smoothed using RMS algorithms with a window width of 150 ms. In accordance with the adopted methodology, the sought value was muscle activity MA (7), which was determined on the basis of the maximum voluntary contraction (MVC) readout measured during the measurement test and during the normalization procedure (MVC_max_). The value of the MVC_max_ signal was the reference value defining the maximum of the tested muscle group in the same static force test for all tested patients. The activity value was determined as the percentage of the measured MVC signal relative to the constant maximum value of MVC_max_ for a given patient. This approach made it possible to compare individual users regardless of what results they achieved during the standardization exercises.

7 $${\text{MA}} = \frac{{{\text{MVC}}}}{{{\text{MVC}}_{{{\text{max}}}} }} \cdot 100\%$$

The performed measurement of the maximum voluntary contraction (MVC) allowed establishing the muscle activity of each of the tested muscles (MA_i_). On the basis of the derived values of MA, the maximal effort of the upper limbs (ME) was determined (8).

8 $${\text{ME}} = \frac{{\mathop \sum \nolimits_{i = 1}^{n} {\text{MA}}_{i} }}{n}$$

where: n—the number of examined muscles, MA_i_—the value of muscular activity of one of the examined muscles.

## Results and discussion

**Figure 9:**
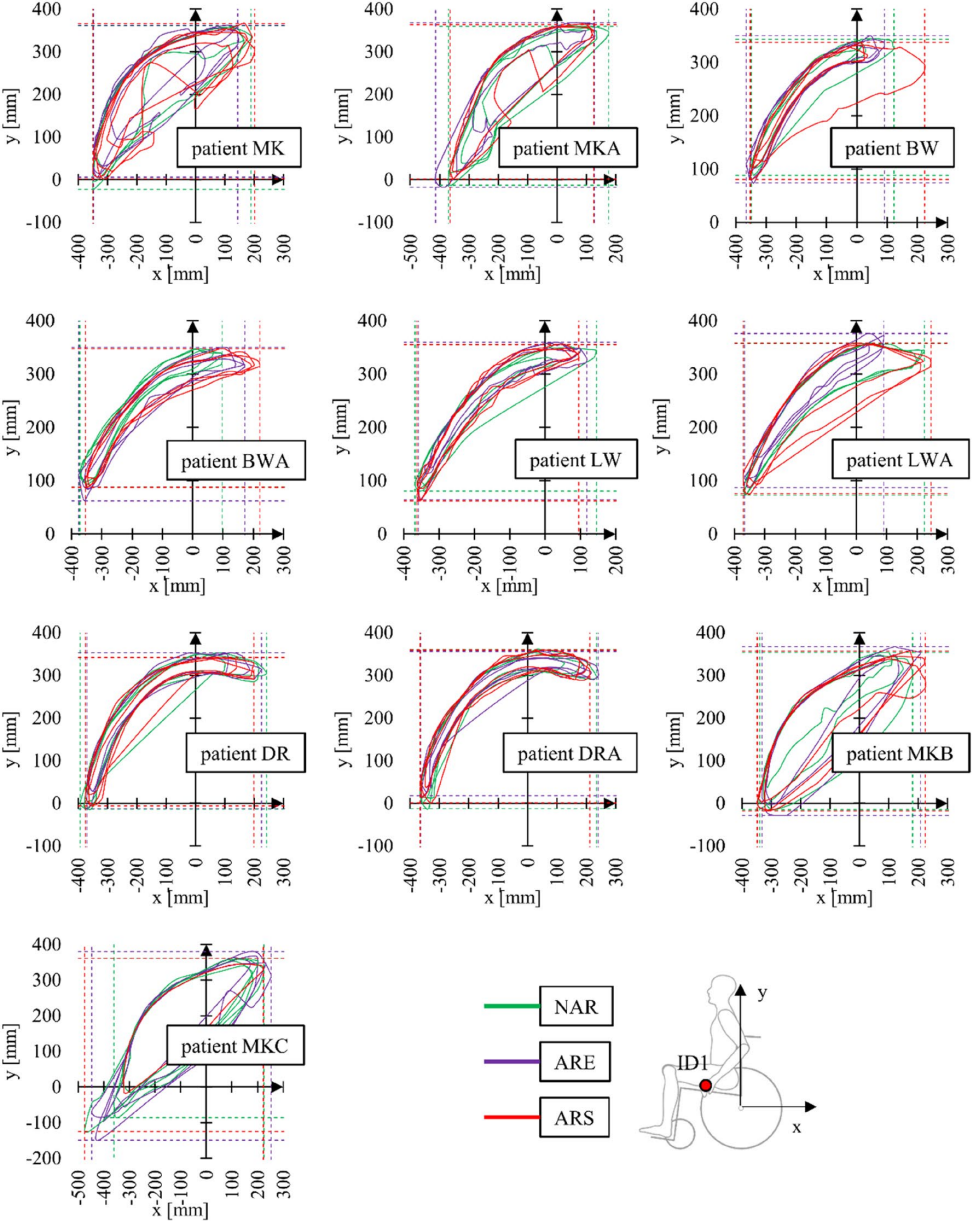
Graphs representing hand position when propelling a wheelchair with no modifications (NAR), with flexible anti-rollback system (ARE) and with stiff anti-rollback system for 10 subjects, where ID1 is the location of the marker on the upper limb.

**Figure 10:**
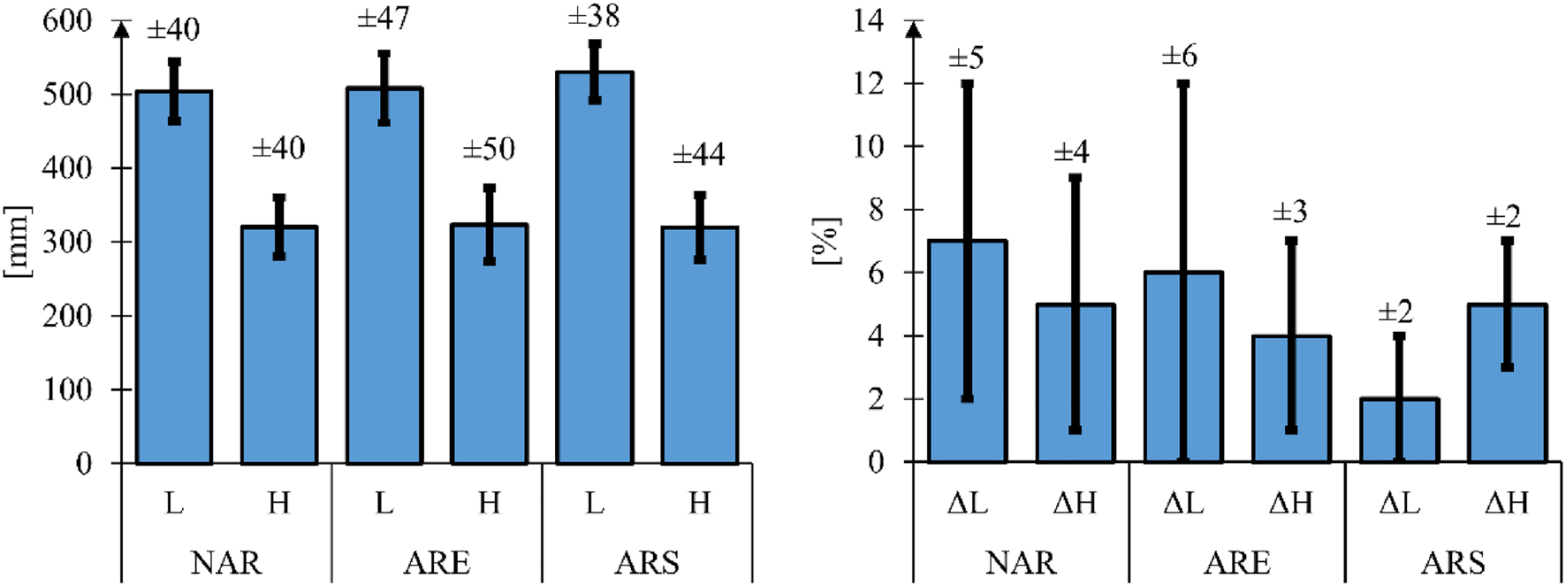
Summary of L and H parameters of the hand position when propelling a wheelchair with no modifications (NAR), with a flexible anti-rollback system (ARE) and with a stiff anti-rollback system—for 10 subjects, where: L—length of the area, H—height of the area, ΔL—% difference in length relative to the largest value for a given subject, ΔH—% difference in height relative to the largest value for a given subject. All mean values were determined at a 95% confidence interval (*p* = 0.05).

In accordance with the adopted research methodology, muscular activity and motion capture measurements were performed simultaneously. First, the measured data was used to determine the hand position during the entire propulsion cycle consisting of the push and return phases. Visual interpretation of the hand position was then presented in the form of graphs representing three measurement tests for each of the tested wheelchair versions and for each of the tested subjects (Fig. 9). After analysis of the registered positions, the average value of the length L and width H of the position for the three wheelchair variants was established for each subject (Fig. 10). In addition, for each subject, the difference in % of the above parameters in relation to the largest of the registered values was determined. The greatest length L was recorded for a wheelchair equipped with a stiff anti-rollback system. As regards the H parameter, the largest value was recorded for a wheelchair with a flexible anti-rollback system.

Differences in the dimensions of the analyzed areas between individual wheelchair types are insignificant. The average values for all subjects are L = 26 mm and H = 4 mm. Due to insignificant variation in the measured parameters, it was assumed that the anti-rollback system variant does not affect the hand trajectory, which in turn does not force the wheelchair user to adopt different body kinematics compared to a conventional wheelchair. Assuming that the differences in the geometric shape of the areas are due to the individual predispositions of the subjects, such as the frequency of push phases and the speed of the wheelchair^41^, the analysis of the hand position during the propulsion cycle was concluded at that point.

**Figure 11:**
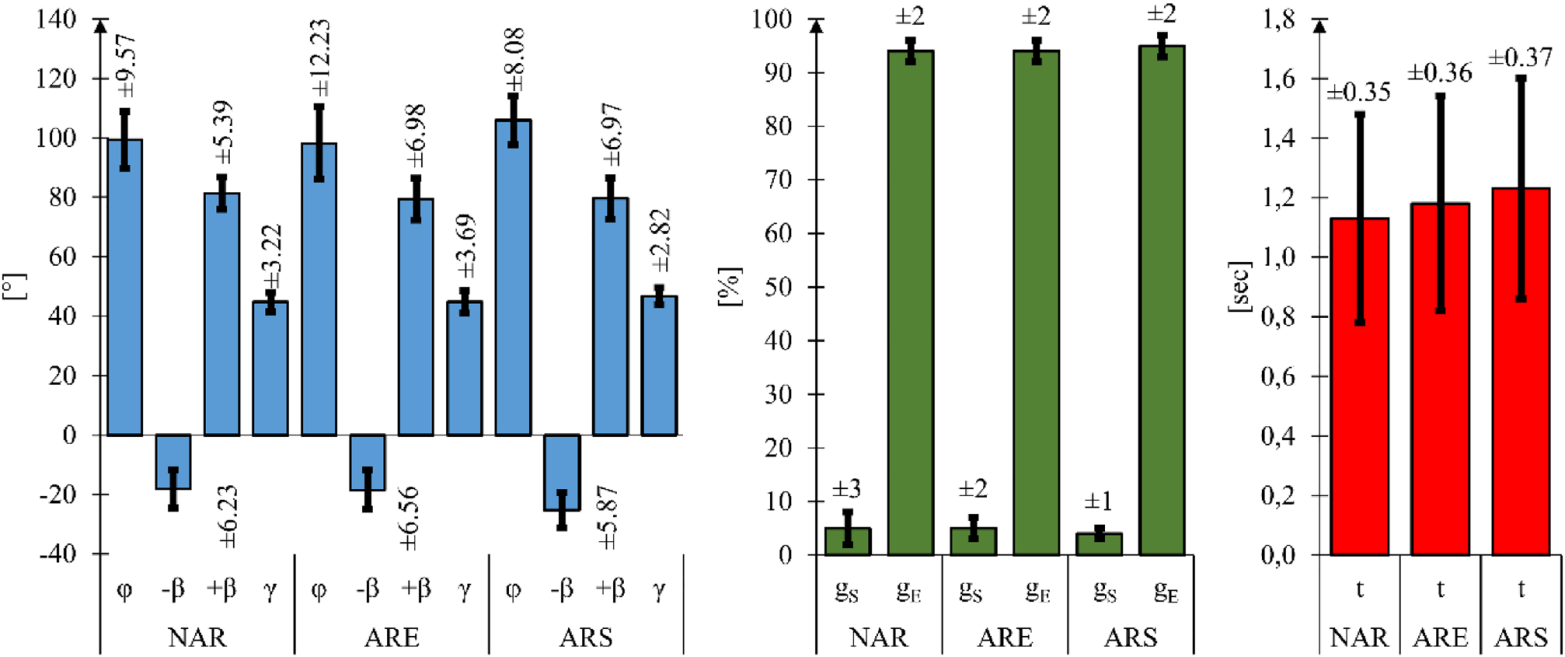
Results of the motion capture measurement analysis of the push phase for the wheelchair without an anti-rollback system (NAR), with a flexible anti-rollback system (ARE) and with a stiff anti-rollback system (ARS). In these graphs: φ—the total push-rim rotation angle, -β—the angular position of the hand on the push-rim at the beginning of the push phase, + β—the angular position of the hand on the push-rim at the end of the push phase, γ—the trend line inclination of the function of the total push-rim rotation angle, t—the duration of the push phase, g_S_—the percentage share of the total push phase duration with the hand gripping the push-rim, g_E_—the percentage share of the total push phase duration with the push-rim released. All mean values were determined at a 95% confidence interval (*p* = 0.05).

Further analysis of the data obtained by the motion capture measurement of the marker placed on the hand consisted in the identification of three individual drive phases for all subjects and all wheelchair variants. The parameters determined from these three samples were averaged and used for further analyses. In order to standardize the time base for all subjects in each sample, the time base for the parameters in question was replaced by the percentage share of the push phase total duration. A similar method of standardizing biomechanical tests conducted on different individuals is used by other researchers, e.g. when measuring muscle activity^42^. Building upon these assumptions, we determined the total rotation angle of the push-rim φ, the angular position of the hand on the push-rim at the beginning of the push phase − β and at the end of it + β, the inclination of the trend line γ of the total push-rim rotation angle increment φ, the duration of the push phase t, the percentage share of the push phase total duration in which the hand was holding the push-rim g_S_ and the percentage share of the push phase in which the hand was released g_E_ (Fig. 11).

Based on the motion capture analysis, it was found that 8 out of the 10 subjects examined tended to rotate the push-rims by the largest angle φ when using a wheelchair equipped with a stiff anti-rollback system. The average total push-rim rotation angle φ for this type of wheelchair was 105.91°. In the case of the wheelchair without the anti-rollback system, that value was 99.39°, while in the case of the wheelchair equipped with the flexible anti-rollback system the value was 98.18°. In analyzing these results, it can be concluded that when using the stiff anti-rollback system, the time when the wheelchair user is transferring muscle power to the propulsion system is longer. This is corroborated by the measurement of the push phase duration. The average value of the push phase t was 1.13 s for the wheelchair without an anti-rollback system, 1.23 s for the wheelchair with a stiff anti-rollback system and 1.18 s for the wheelchair with a flexible anti-rollback system. On the basis of these findings, one can notice an evident correlation between the use of ramp assist systems and the increase in duration of the push phase t. In the case of a wheelchair equipped with a stiff anti-rollback system, despite the largest value of the total push-rim rotation angle (φ) and the longest duration of the push phase, the highest rate of total push-rim rotation angle increment (γ) was observed. This rate was determined by the trend line inclination of the function of the total push-rim rotation angle change. The value of this angle was 44.76° for the wheelchair without an anti-rollback system, 46.70° for the wheelchair with a stiff anti-rollback system and 44.77° for the wheelchair with a flexible anti-rollback system.

Regarding hand kinematics, we found that when using the wheelchair with the stiff anti-rollback system, at the beginning of the push phase, the position of the hand was furthest back. Its angular position on the push-rim − β averaged − 25.23° for all subjects. In the case of the wheelchair without the anti-rollback system, that value was − 18,16°, while in the case of the wheelchair equipped with the flexible anti-rollback system it was − 18.48°. In the case of the angular position of the hand on the push-rim at the end of the push phase (+β), there were no significant differences among the different anti-rollback systems. In the case of the wheelchair without the anti-rollback system, the + β angle was 81.33°, while for the wheelchairs with the stiff and flexible anti-rollback systems, these values were 79.66° and 79.33°, respectively. The analysis of the push phase stages with the hand gripping the push-rim g_S_ and with the push-rim released g_E_ did not show any impact of the ramp assist systems on these values. This was expected due to the users' individual propulsion styles^43,44^.

**Figure 12:**
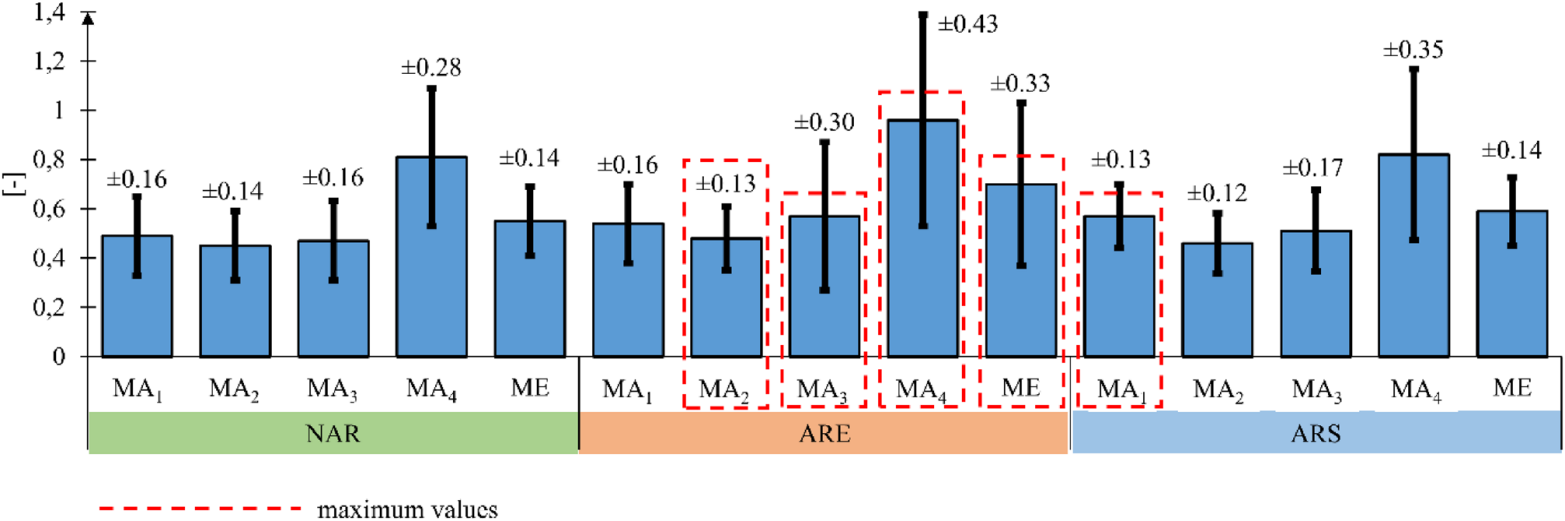
Results of the maximum effort (ME) and maximum muscle activity (MA) analysis for the wheelchair without the anti-rollback system (NAR), with the flexible anti-rollback system (ARE) and with the stiff anti-rollback system (ARS), where: MA_1_—maximum value of muscular activity for the deltoid muscle anterior, MA_2_—maximum value of muscular activity for the deltoid muscle posterior, MA_3_—maximum value of muscular activity for the triceps brachii muscle, MA_4_—maximum value of muscular activity for the extensor carpi radialis longus, ME—maximum value of total upper limb effort. All mean values were determined at the 95% confidence interval (*p* = 0.05).

Muscle activity measurement for each of the 10 subjects was performed in triplicate for each of the three wheelchair variants. The average muscular effort of each muscle expressed as a function of the percentage share of the total push phase duration was then determined for all the three measurement tests (Fig. 11) On the basis of this data, an analysis was performed to assess the maximum muscular effort of each of the examined muscles and the overall maximum muscular effort of the entire upper limb ME (Fig. 12).

**Figure 13:**
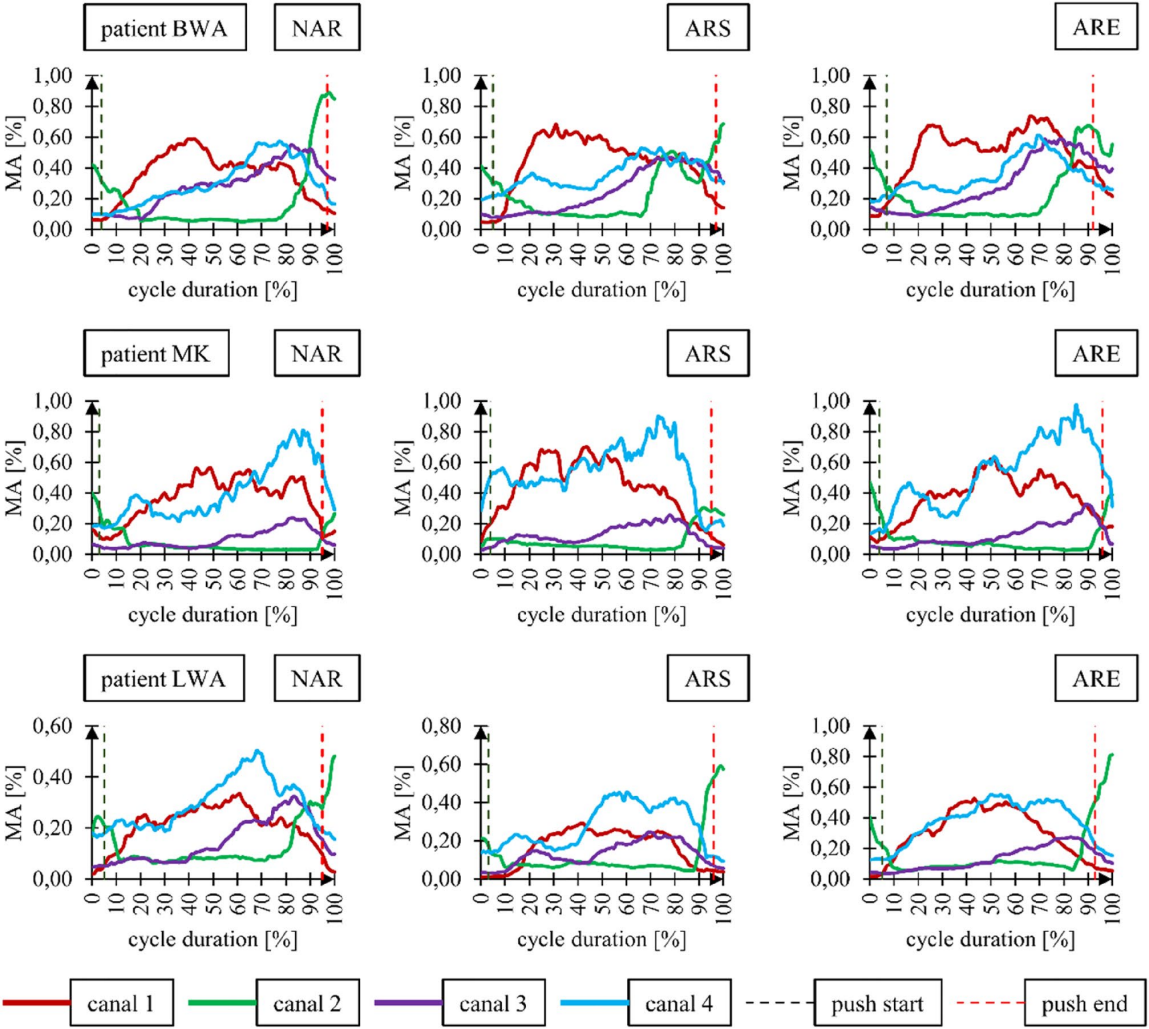
Graphs for selected subjects representing the muscular activity of four muscles as a function of the percentage share of the total push phase duration. In these graphs: NAR—wheelchair without the anti-rollback system, ARS—wheelchair with the stiff anti-rollback system, ARE—wheelchair with the flexible anti-rollback system, canal 1—deltoid muscle anterior, canal 2—deltoid muscle posterior, canal 3—triceps brachii, canal 4—extensor carpi radialis longus.

An analysis of the EMG signal for individual subjects showed that neither the presence nor type of ramp assist system used affected the shape of the muscular activity function. This is illustrated in Fig. 13 for three randomly selected subjects. No changes in the shape of muscular effort curves indicate that ramp assist systems do not affect the propulsion style and the muscles used. In deriving the maximum muscular effort values, we observed that these values tended to be the lowest for the wheelchair without the anti-rollback system. The average maximum muscular effort (ME) of the whole upper limb was 0.55 ± 0.19 for the wheelchair without an anti-rollback system, 0.59 ± 0.19 for the wheelchair with a stiff anti-rollback system and 0.70 ± 0.46 for the wheelchair with a flexible anti-rollback system. On analyzing the standard deviation, the largest discrepancy was observed for the flexible anti-rollback system. This may indicate that this particular design has an impact on the subjective sensation of the user, which determines the propulsion style and transmission mode of driving force. Similar values of maximum ME were observed regardless of the type of wheelchair used for the following muscles: Deltoid muscle anterior (canal 1) and posterior (canal 2), Triceps brachii (canal 3). The largest differences in maximum ME ranging from 0.81 to 0.96 were found for the extensor carpi radialis longus muscle (canal 4). This muscle was involved in gripping the push-rims, and the flexible anti-rollback system compelled 9 out of 10 users to apply a stronger hand grip on the rims. This is particularly true in the later stages of the push phase (50–90%) (Fig. 13).

**Figure 14:**
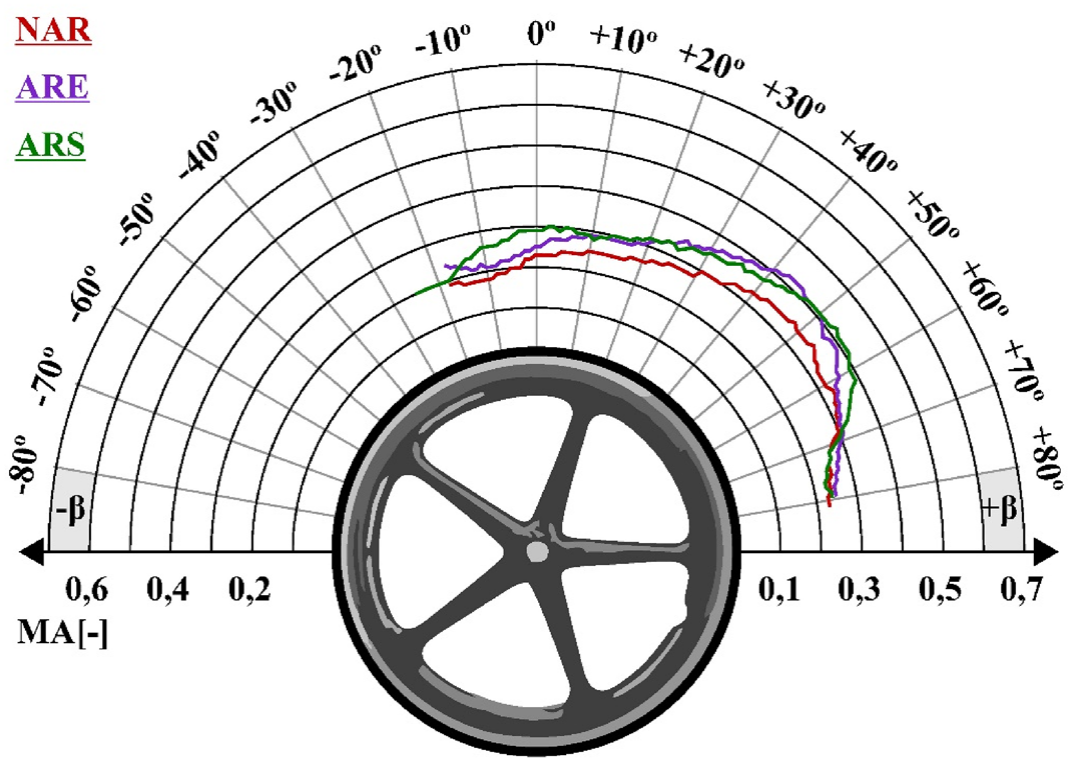
Graphs of the average total muscular effort of the upper limb as a function of its position on the push-rim. In the graphs: NAR—wheelchair without an anti-rollback system, ARS—wheelchair with a stiff anti-rollback system, ARE—wheelchair with a flexible anti-rollback system.

The main assumption of this research was to combine motion capture and EMG measurements in order to assess two variants of anti-rollback system. Using a common basis for both measurement signals, namely, the percentage share of the total push phase duration, graphs of total upper limb muscular effort were made for each of the 10 subjects depending on the angle of the hand position on the push-rims (Fig. S1-S10). The average graph of total upper limb muscular effort for 10 subjects as a function of time is presented in Fig. 14.

Assessment of the combined EMG and motion capture signals showed that the highest intensity of total muscular effort for the upper limb was recorded for the hand holding the push-rims in a position between 30° and 70°. What is more, the lowest maximum muscular effort of the whole upper limb was recorded for the wheelchair without any modifications. In the case of the wheelchair with the stiff anti-rollback system, the difference was 0.04, while for the wheelchair with the flexible anti-rollback system it was 0.16. It should be stressed that for the flexible anti-rollback system, the highest values in relation to the other solutions were achieved only for hand positions from ~ 25° to 50°. For the remaining angles, the muscular effort of the whole upper limb was lower compared to the stiff anti-rollback system.

## Conclusions

Climbing ramps is one of the most difficult and dangerous activities performed by wheelchair users. The existing research describes the possibility of using ramp assist systems and their qualitative assessment in terms of safety improvement^5,45,46^. In this paper, a quantitative evaluation of the anti-rollback system in two different variants was performed. Further stages of research and development will establish new directions for the designs of ramp assist systems.

The motion capture measurements conducted have shown that the push phase is the longest when using a wheelchair with the stiff anti-rollback system. The average time for this solution was 1.23 s. Increasing the duration of the push phase is desirable as it positively affects the value of the total push-rim rotation angle φ and translates into uniform load on the muscular system. In addition, extending the duration of the push phase helps in the process of learning to use a wheelchair^47^. Increasing the total rotation angle was also accompanied by the highest rate of angle increment as a function of time γ. The observed increase in angle increment rate translates into a higher speed of the wheelchair. This, in turn, positively affects the generated driving force^48^. In the case of the wheelchair with the stiff anti-rollback system, we also observed that the user tended to grip the push-rims earlier. This was represented by the angular position of the hand β, which for this particular technical solution ranged from − 25° to 80°. As regards the percentage share of the total push phase duration with the hand gripping the push-rim (g_S_) and the push-rim released (g_E_), no impact of the ramp assist system was observed in this regard. The values for these points were ~ 5% and ~ 95% respectively.

The EMG measurement indicated that the user exerts slightly more muscular effort when using wheelchairs with anti-rollback systems no matter what type of system. Moreover, the maximum momentary exertion for the whole upper limb was the greatest when using the flexible anti-rollback system. It should be noted, however, that the maximum values were obtained only for specific hand positions of 30° to 50°. For the other hand positions, the measured values of the whole upper limb effort were lower than in the case of the stiff anti-rollback wheelchair. Still, in analyzing the total upper limb muscular effort as a whole, the values obtained are similar (Fig. 7). Significantly lower muscular effort was recorded only in the case of the wheelchair without any modifications. This could be expected as equipping the wheelchair with an additional roller coupled with the drive wheel introduces additional motion resistance. This resistance must be compensated for by the propulsion force generated by the upper limb, which translates into an increased muscular effort. At the same time, it is true that equipping the wheelchair with an anti-rollback system increases the overall effort of the user. This is due to the introduction of more elements into the system, which primarily generates additional friction and elastic deformation in the system. Therefore, a solution that allows for enabling or disabling the anti-rollback system, depending on the user's needs, should be considered particularly advantageous. From an energy efficiency point of view, the use of this system is unjustified on a non-sloping surface.

In assessing the determined parameters, we concluded that despite the increase in muscular effort, the use of an anti-rollback system is beneficial for the wheelchair user, due to greater safety, which is the most important criterion. Furthermore, the increase in muscular effort is not significant enough to be harmful to the human muscular system. A particularly desirable feature of the anti-rollback system is the increase in the duration of the push phase. It allows the user to maintain a constant contraction rather than flexing the muscles abruptly. This translates into a reduced risk of injury^49^. Another argument in favor of the anti-rollback system is that it gives the wheelchair user the opportunity to rest and take a break from propulsion, even when moving uphill. This possibility significantly increases the physical and psychological comfort compared to a conventional wheelchair.

The research results show that the use of the stiff anti-rollback system involves the same muscles, to a similar extent, as in the case of the conventional wheelchair. In turn, the flexible anti-rollback system allows for new possibilities in terms of the speed and intensity of the generated propulsion force. This is confirmed by the largest discrepancies in the results obtained for this system. It should be underlined, however, that these tests were carried out on a track with an even, smooth surface. This might be a reason for the lack of differences between results for the stiff and flexible anti-rollback system. Further research will focus on tests on different types of surfaces, which will allow assessing the impact of the elastic body that presses the anti-rollback system against the drive wheel. We also plan to conduct tests for different diameters of the roller, which will affect the deformation of the wheelchair tire during frictional coupling. Additional research will also be carried out on the wheelchair dynamics to determine the ratio of the driving torque delivered to the system to the generated muscular activity.

Based on the above observations and analyzes, we found that of the two proposed concepts of anti-rollback system, the rigid variant is more advantageous. This choice is motivated by the lack of significant differences in the kinematics of the upper limb, a noticeable difference in muscle effort (ME) and the difference in the complexity of the structure. In the case of the anti-rollback system in the rigid variant, muscle effort (ME) similar to a wheelchair without modification (NAR) was measured and, additionally, was significantly lower than the anti-rollback system in the flexible variant (ARE). The design of the anti-rollback system in the rigid variant is very simple. It consists of three basic elements and, as shown in the example embodiment, it can replace the original wheelchair parking brake while maintaining the ability to be enabled of disabled depending on the user's needs.

## Supplementary Information

Supplementary Information.

## Data Availability

All data generated or analysed during this study are included in this published article and its supplementary information files.
