# Supplementary material for: Evaluation of anti-rollback systems in manual wheelchairs: muscular activity and upper limb kinematics during propulsion
Source: Sci Rep. 2022 Nov 9;12:19061. doi: 10.1038/s41598-022-21806-z (PMC9646883; doi:10.1038/s41598-022-21806-z)
Supplement: Supplementary file 1 — Supplementary Information. [file 41598_2022_21806_MOESM1_ESM.docx]

**Supplementary Info**

**Appendix A**


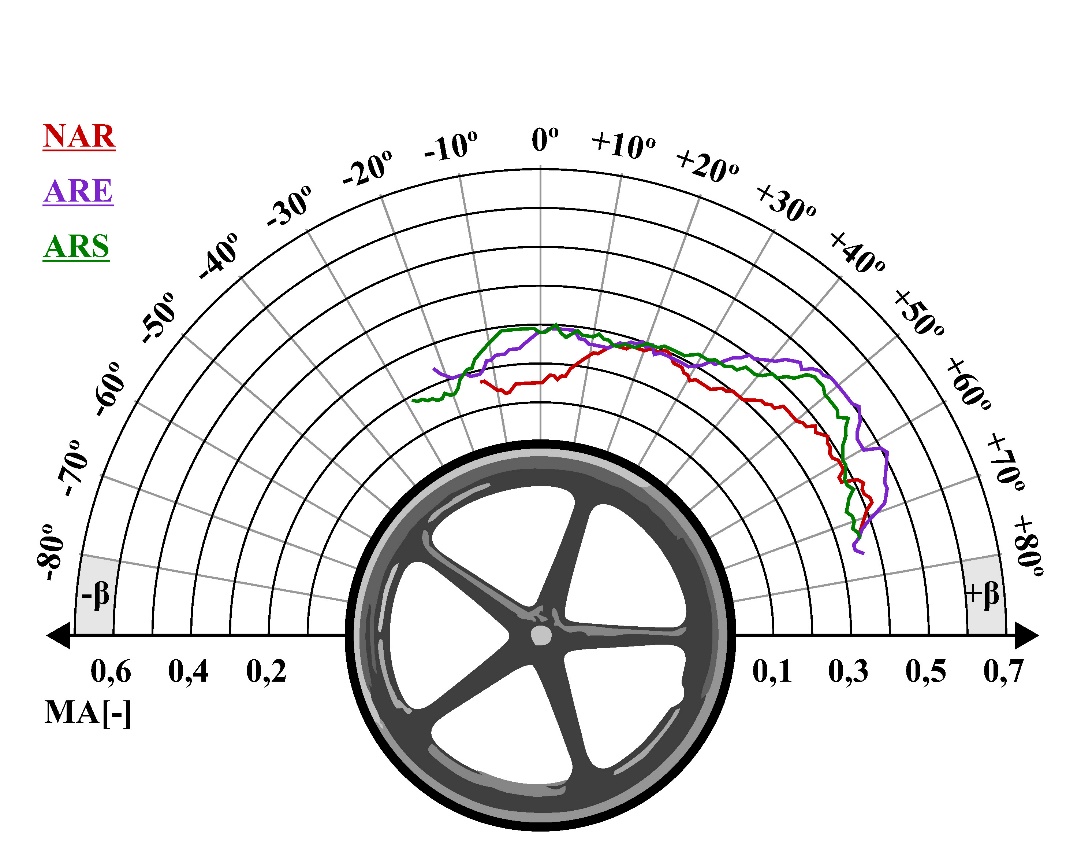


Fig. S1. Graphs representing total muscular effort of the upper limb as a function of its position on the pushrim for subject BWA. Where: NAR – wheelchair without anti-rollback system, ARS – wheelchair with stiff anti-rollback system, ARE – wheelchair with flexible anti-rollback system


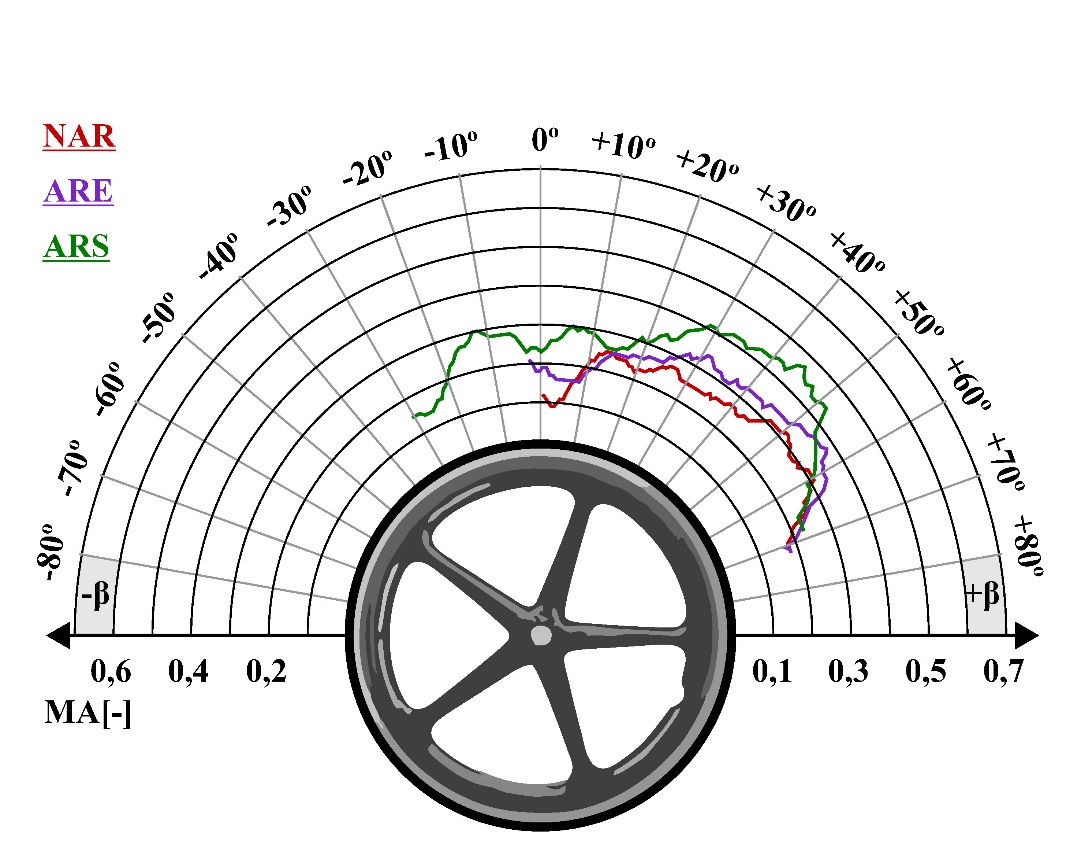


Fig. S2. Graphs representing total muscular effort of the upper limb as a function of its position on the pushrim for subject BW. Where: NAR – wheelchair without anti-rollback system, ARS – wheelchair with stiff anti-rollback system, ARE – wheelchair with flexible anti-rollback system


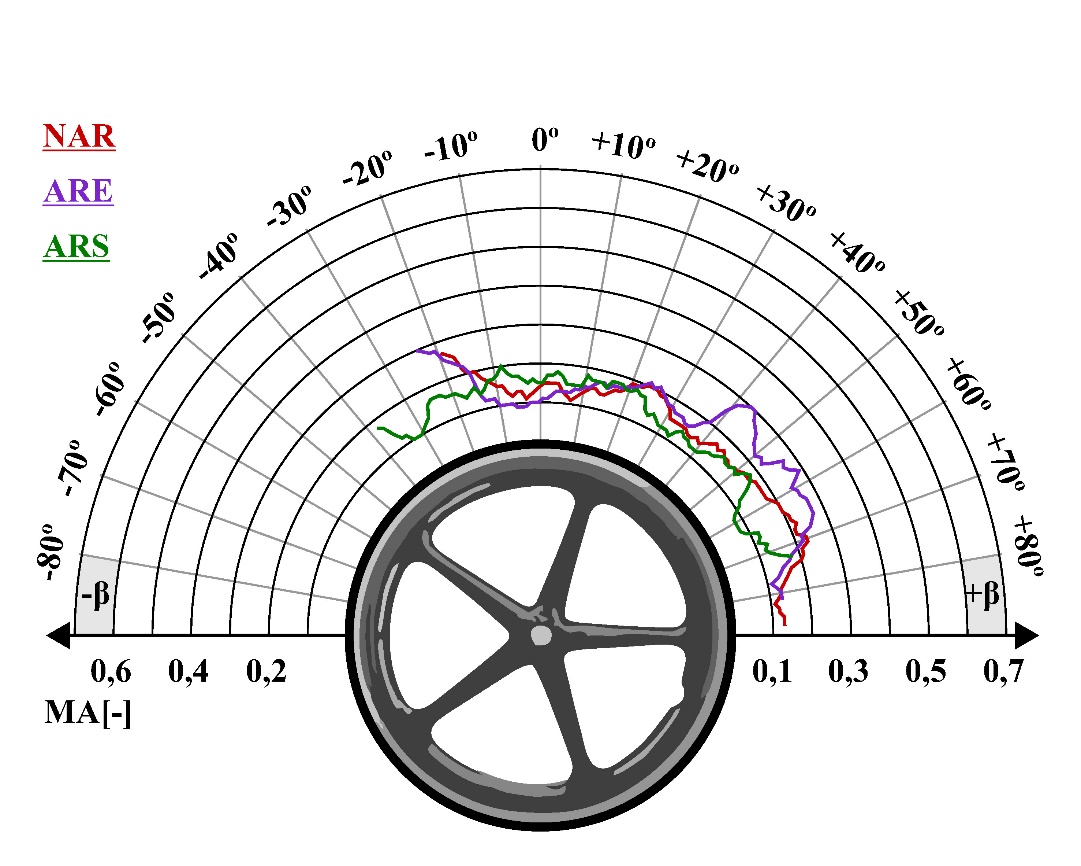


Fig. S3. Graphs representing total muscular effort of the upper limb as a function of its position on the pushrim for subject DRA. Where: NAR – wheelchair without anti-rollback system, ARS – wheelchair with stiff anti-rollback system, ARE – wheelchair with flexible anti-rollback system


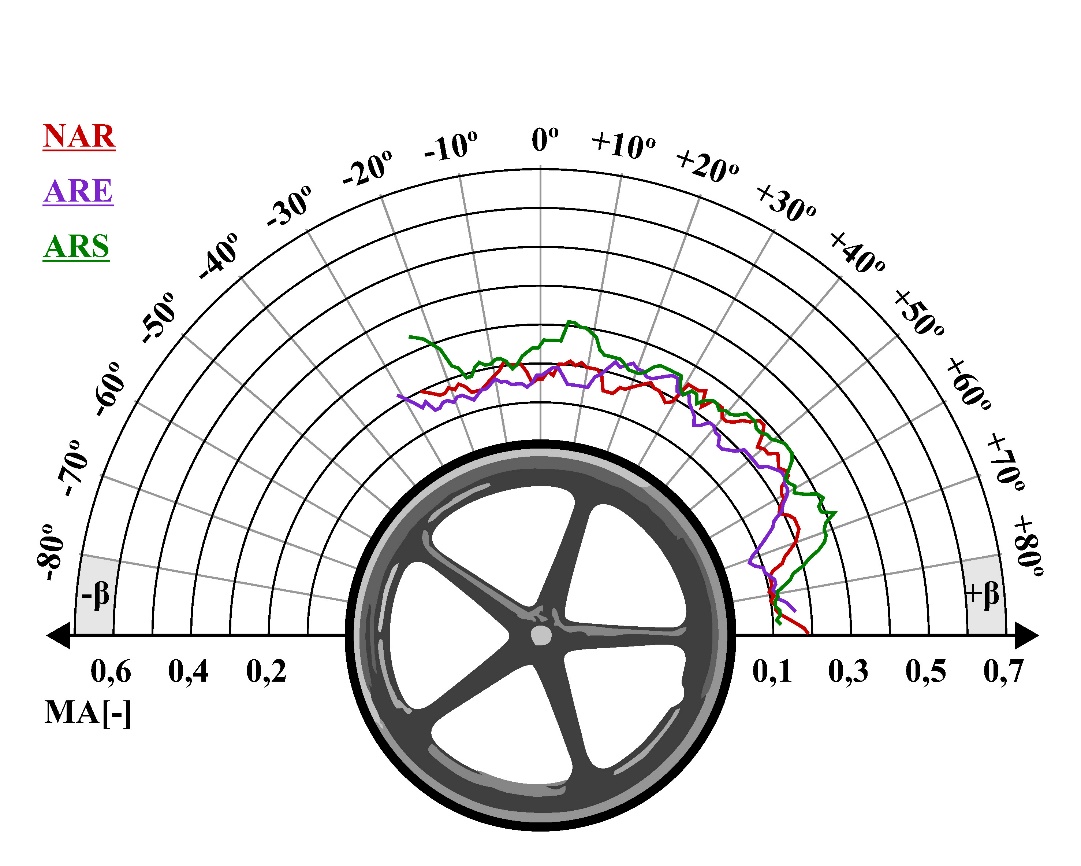


Fig. S4. Graphs representing total muscular effort of the upper limb as a function of its position on the pushrim for subject DR. Where: NAR – wheelchair without anti-rollback system, ARS – wheelchair with stiff anti-rollback system, ARE – wheelchair with flexible anti-rollback system


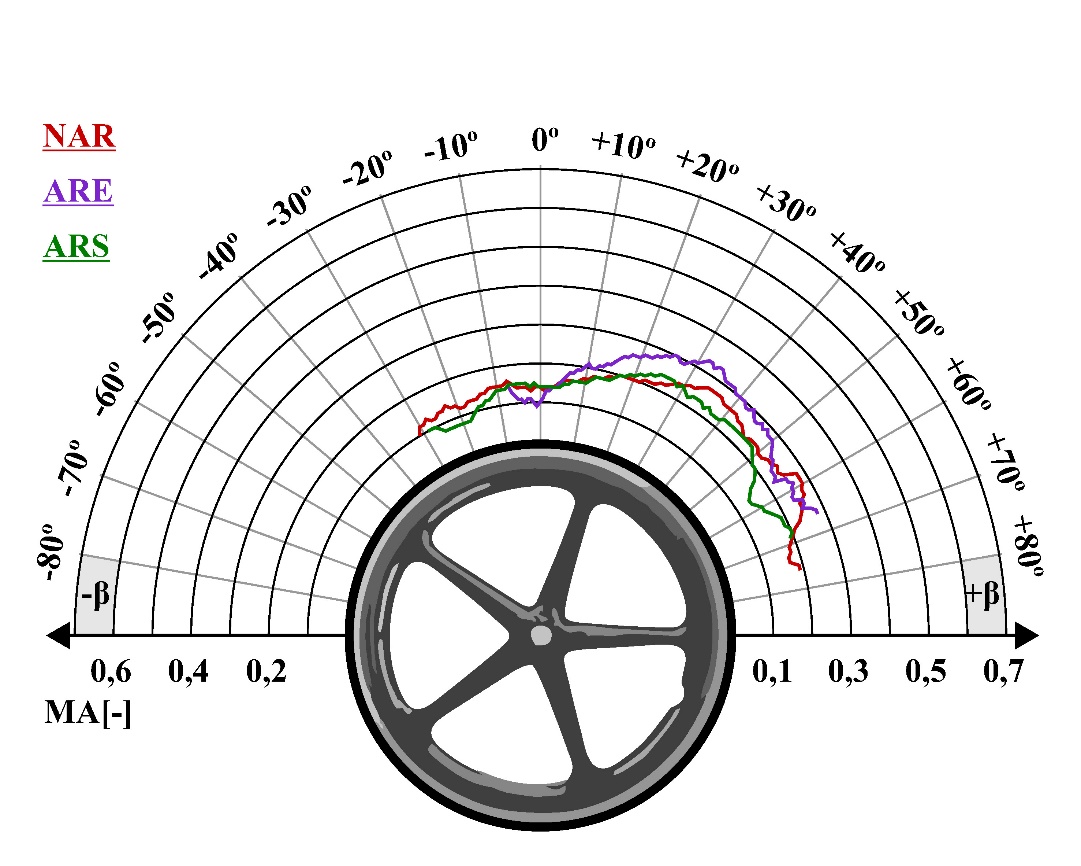


Fig. S5. Graphs representing total muscular effort of the upper limb as a function of its position on the pushrim for subject LWA. Where: NAR – wheelchair without anti-rollback system, ARS – wheelchair with stiff anti-rollback system, ARE – wheelchair with flexible anti-rollback system


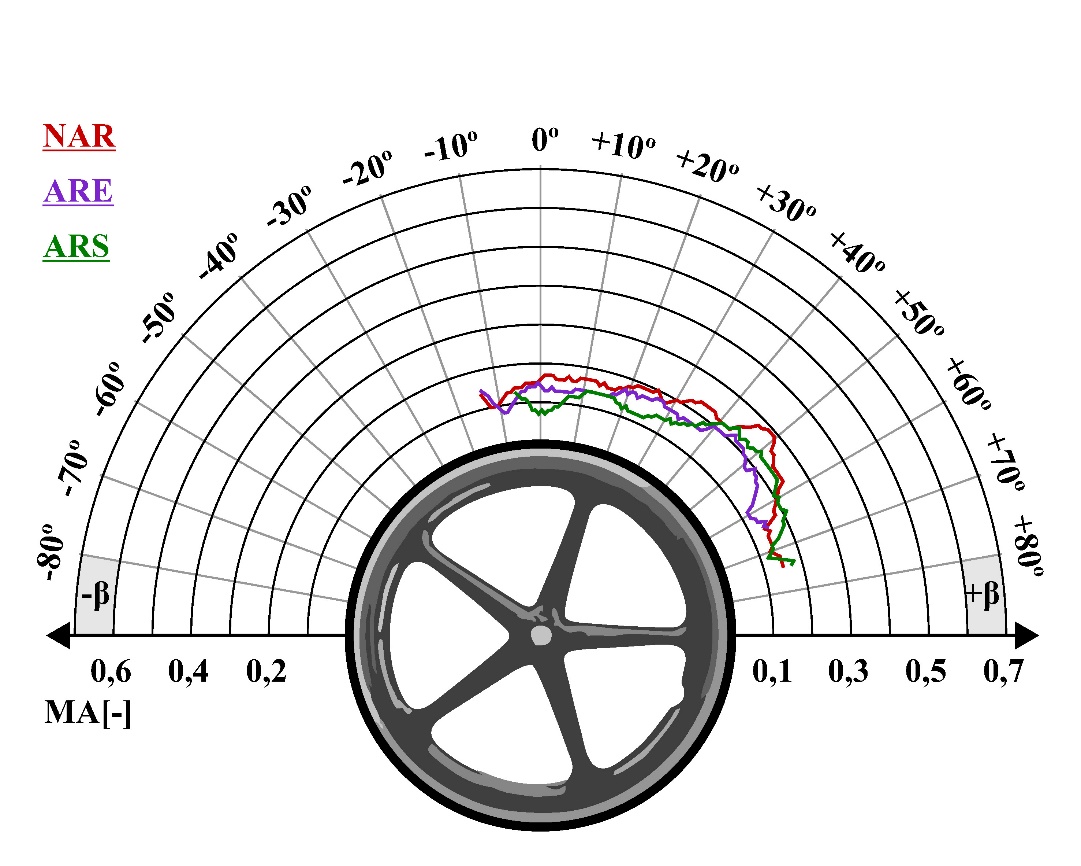


Fig. S6. Graphs representing total muscular effort of the upper limb as a function of its position on the pushrim for subject LW. Where: NAR – wheelchair without anti-rollback system, ARS – wheelchair with stiff anti-rollback system, ARE – wheelchair with flexible anti-rollback system


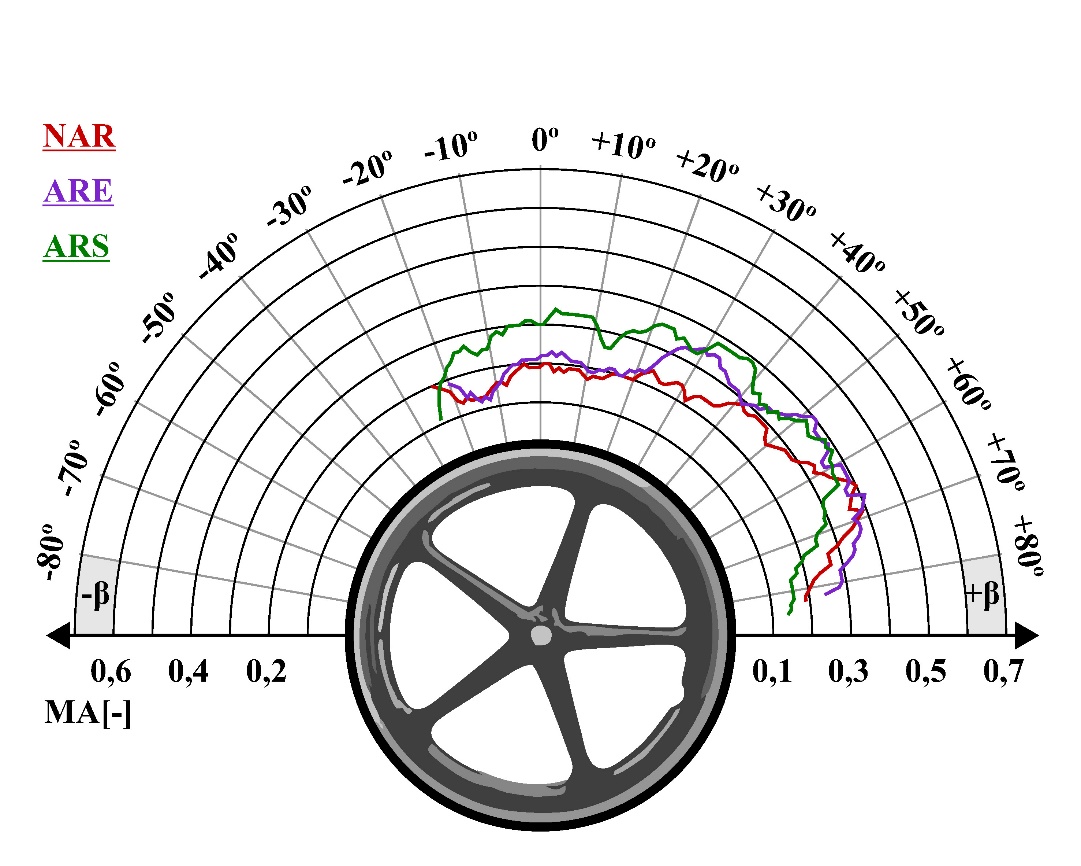


Fig. S7. Graphs representing total muscular effort of the upper limb as a function of its position on the pushrim for subject MK. Where: NAR – wheelchair without anti-rollback system, ARS – wheelchair with stiff anti-rollback system, ARE – wheelchair with flexible anti-rollback system


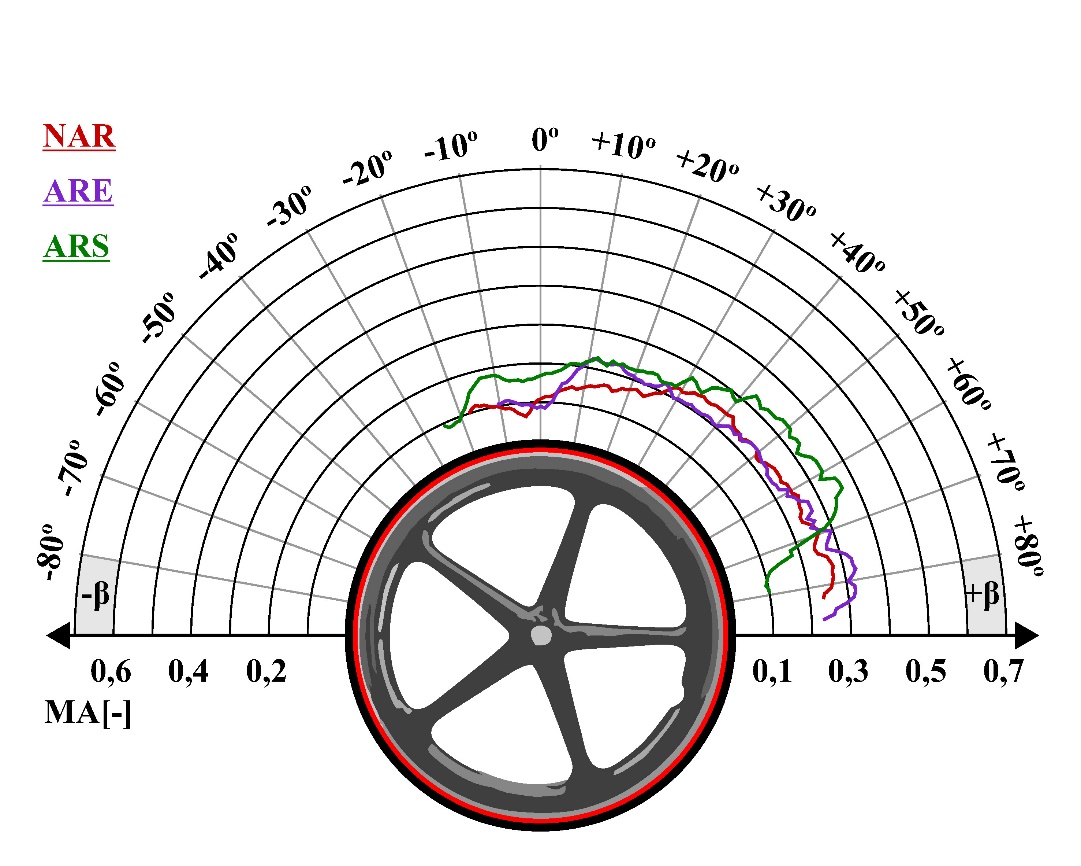


Fig. S8. Graphs representing total muscular effort of the upper limb as a function of its position on the pushrim for subject MKA. Where: NAR – wheelchair without anti-rollback system, ARS – wheelchair with stiff anti-rollback system, ARE – wheelchair with flexible anti-rollback system


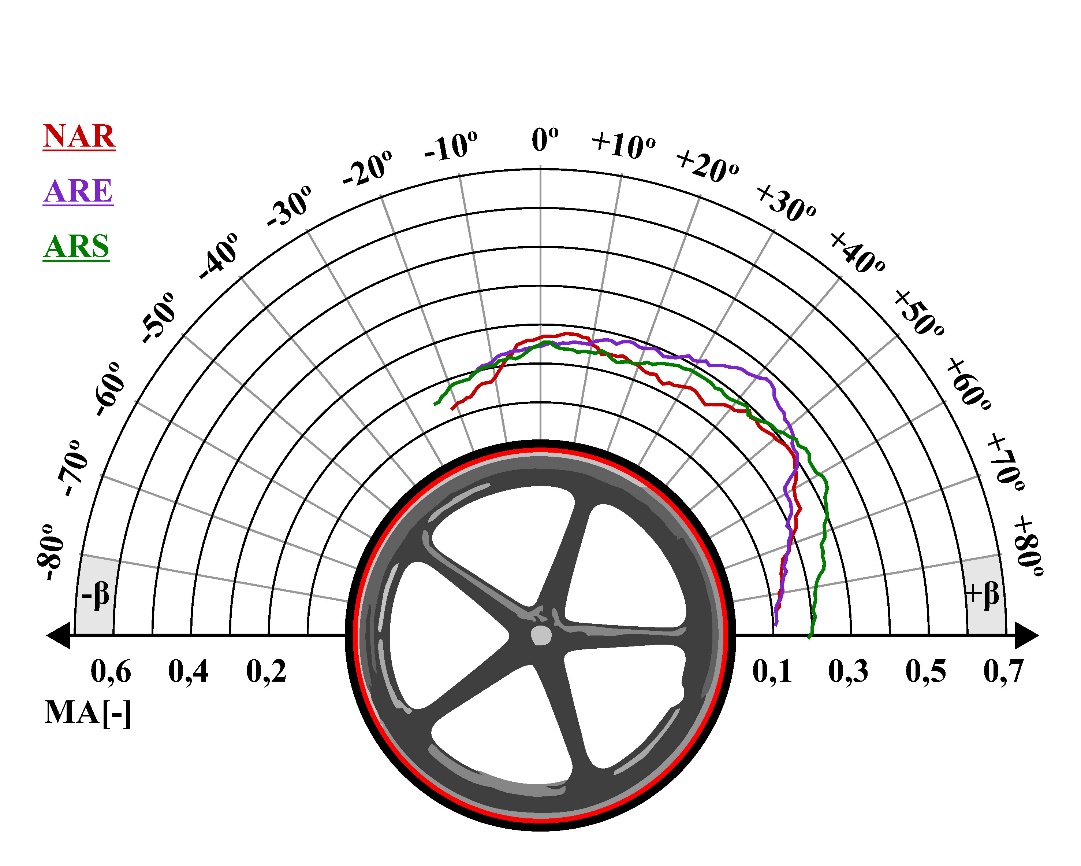


Fig. S9. Graphs representing total muscular effort of the upper limb as a function of its position on the pushrim for subject MKB. Where: NAR – wheelchair without anti-rollback system, ARS – wheelchair with stiff anti-rollback system, ARE – wheelchair with flexible anti-rollback system


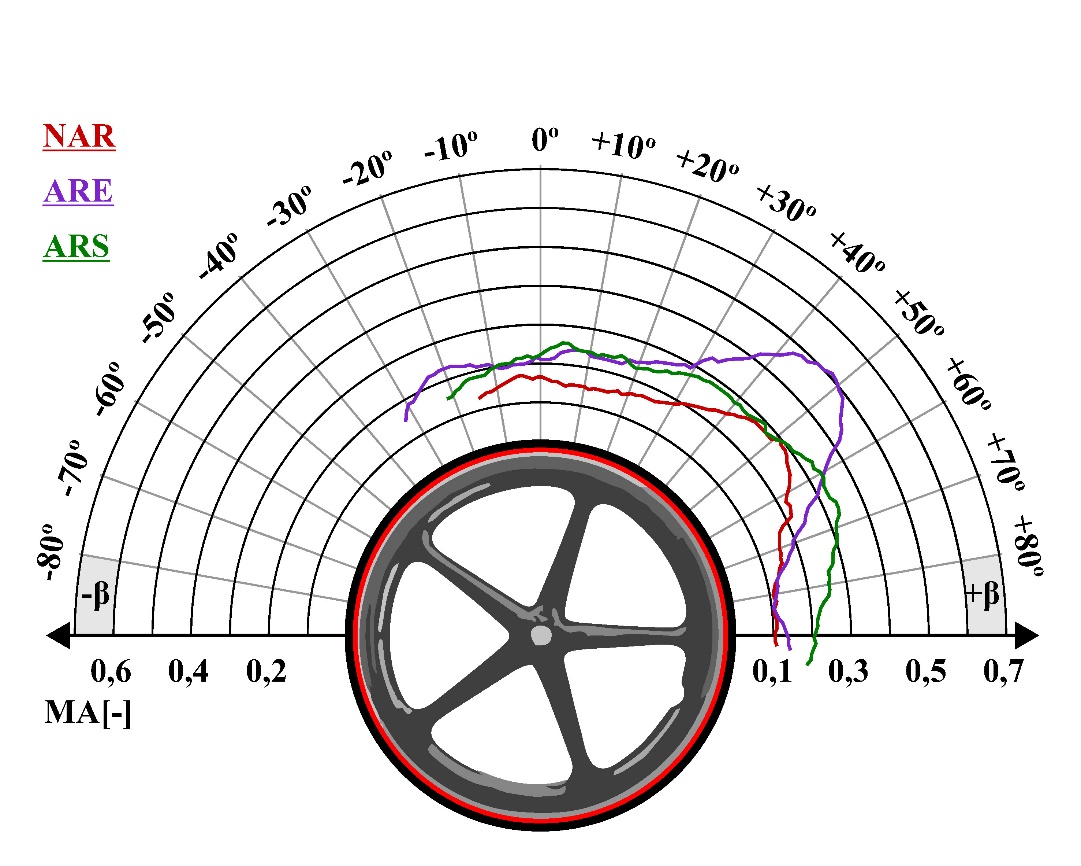


Fig. S10. Graphs representing total muscular effort of the upper limb as a function of its position on the pushrim for subject MKC. Where: NAR – wheelchair without anti-rollback system, ARS – wheelchair with stiff anti-rollback system, ARE – wheelchair with flexible anti-rollback system
